# Chitinase A, a tightly regulated virulence factor of *Salmonella enterica* serovar Typhimurium, is actively secreted by a Type 10 Secretion System

**DOI:** 10.1371/journal.ppat.1011306

**Published:** 2023-04-05

**Authors:** Lena Krone, Larissa Faass, Martina Hauke, Christine Josenhans, Tobias Geiger

**Affiliations:** 1 Max von Pettenkofer-Institute, Chair for Medical Microbiology and Hygiene, Ludwig Maximilian University (LMU), Munich, Germany; 2 Deutsches Zentrum für Infektionsforschung, partner site Munich, Germany; University of California Davis School of Medicine, UNITED STATES

## Abstract

As a facultative intracellular pathogen, *Salmonella enterica* serovar Typhimurium is one of the leading causes of food-borne diseases in humans. With the ingestion of fecal contaminated food or water, *S*. Typhimurium reaches the intestine. Here, the pathogen efficiently invades intestinal epithelial cells of the mucosal epithelium by the use of multiple virulence factors. Recently, chitinases have been described as emerging virulence factors of *S*. Typhimurium that contribute to the attachment and invasion of the intestinal epithelium, prevent immune activation, and modulate the host glycome. Here we find that the deletion of *chiA* leads to diminished adhesion and invasion of polarized intestinal epithelial cells (IEC) compared to wild-type *S*. Typhimurium. Interestingly, no apparent impact on interaction was detected when using non-polarized IEC or HeLa epithelial cells. In concordance, we demonstrate that *chiA* gene and ChiA protein expression was solely induced when bacteria gain contact with polarized IEC. The induction of *chiA* transcripts needs the specific activity of transcriptional regulator ChiR, which is co-localized with *chiA* in the chitinase operon. Moreover, we established that after *chiA* is induced, a major portion of the bacterial population expresses *chiA*, analyzed by flow cytometry. Once expressed, we found ChiA in the bacterial supernatants using Western blot analyses. ChiA secretion was completely abolished when accessory genes within the chitinase operon encoding for a holin and a peptidoglycan hydrolase were deleted. Holins, peptidoglycan hydrolases, and large extracellular enzymes in close proximity have been described as components of the bacterial holin/peptidoglycan hydrolase-dependent protein secretion system or Type 10 Secretion System. Overall, our results confirm that chitinase A is an important virulence factor, tightly regulated by ChiR, that promotes adhesion and invasion upon contact with polarized IEC and is likely secreted by a Type 10 Secretion System (T10SS).

## Author summary

Being considered as one of the key global causes of diarrheal diseases, *Salmonella* can cause diseases from mild salmonellosis to life-threatening diseases depending on host factors and the serotypes of *Salmonella*. Within the species *Salmonella enterica*, serovar Typhimurium is the most important human food-borne bacterial pathogen contracted through the consumption of contaminated food or drinking water. Highly adapted, *S*. Typhimurium owns a plethora of tightly regulated virulence factors, which when needed, are secreted by specific secretion systems to facilitate bacterial infection. Besides classical virulence factors such as toxins, *S*. Typhimurium also takes advantage of metabolic enzymes degrading various substrates important for colonization. Here, we demonstrate that chitinase A produced by *S*. Typhimurium is specifically induced and promotes adhesion and invasion of polarized intestinal epithelial cells. The gene encoding for chitinase A is organized within a defined operon that includes three other important genes. One encodes for the transcriptional regulator ChiR, which we show is essential for the induction of chitinase A expression upon contact with polarized intestinal epithelial cells. The other two encode for a holin and a peptidoglycan hydrolase, components of a Type 10 Secretion System, which is responsible for the active secretion of chitinase A in *S*. Typhimurium.

## Introduction

*Salmonella enterica* is one of the most common food-borne pathogens and is responsible for hundreds of thousands of deaths worldwide, particularly in developing countries [1]. Some serovars, such as *Salmonella enterica* serovar Typhi (*S*. Typhi) and serovar Paratyphi A (*S*. Paratyphi A) are strictly human host-adapted, where they cause a systemic infection with symptoms known as typhoid or paratyphoid fever [2,3]. Other serovars, such as *Salmonella enterica* serovar Typhimurium (*S*. Typhimurium) have a broad host range. These "generalists" most often cause self-limiting infections that remain confined to the gastrointestinal tract in healthy humans [4]. Ingested by contaminated food or drinking water *Salmonella* reaches the distal portion of the ileum, where it is thought most of the replication takes place [5]. Here, by the use of its flagella and chemotactic system, *Salmonella* overcomes the protective mucus layer, composed of mucin glycoproteins that covers the intestinal epithelium [6–8]. The subsequent adhesion and invasion of the intestinal epithelial cells (IEC) are mediated by various fimbrial and nonfimbrial adhesion factors, and the Type 3 protein secretion system (T3SS-1) [9–11]. By contact with the host cell, the T3SS-1 delivers bacterial effector proteins into the host cell, which modulate various host processes ultimately resulting in the internalization of *Salmonella*. Besides the well-known T3SS, other protein secretion systems exist in *Salmonella enterica*. Recently, a novel protein secretion system, responsible for the secretion of typhoid toxin, has been described in *S*. Typhi [12,13]. In this system, the secretion mechanism differs significantly from complex multiprotein secretion machineries such as the T3SS. The central components are a holin inner membrane protein in concert with a specialized peptidoglycan hydrolase. That peptidoglycan hydrolase, once translocated to the periplasm by the holin, facilitates the secretion of typhoid toxin by cleaving the peptidoglycan layer. Genomic analyses have indicated homologous secretion systems, named holin/peptidoglycan hydrolase-dependent protein secretion systems or Type 10 Secretion Systems (T10SS) [14] in a range of bacteria possessing hydrolytic enzymes, holin membrane proteins, and substrate proteins in close vicinity [12,14]. In the Gram-negative opportunistic pathogen, *Serratia marcescens* comprehensive studies have shown that the T10SS facilitates the secretion of chitinases [15–17]. Chitinases are virulence factors in many pathogenic bacteria, important for the colonization of organs [18,19], attachment and invasion of host cells [20–22], promoting intracellular survival [23,24], and modulating the host immune response [25]. For *S*. Typhimurium and *S*. Typhi, a contribution of chitinases to the pathogenicity of both serovars has been shown very recently [26,27]. Chitinase-dependent remodeling and interaction with surface glycans promote the adhesion and invasion of intestinal epithelial cells (IEC) by *Salmonella*. In vivo studies in mice showed that chitinases promote *Salmonella* attachment to the intestinal epithelium, the invasion of the intestine, and enhanced dissemination to other organs [26,27].

In this study, we verified the importance of ChiA as a virulence factor for *S*. Typhimurium. It significantly impacts the adherence and invasion competency of *S*. Typhimurium in contact with human intestinal epithelial cells (IEC). Interestingly, impaired adherence and invasion of a Δ*chiA* mutant compared to wild-type *S*. Typhimurium could only be detected when infecting polarized IEC. This was associated with strongly induced ChiA expression upon contact exclusively with polarized IEC. Expression analyses also indicate a positive but stringent regulation of *chiA* by a transcriptional regulator ChiR, encoded within the chitinase operon. This regulator is also strongly induced upon contact with polarized IEC only. Moreover, we show that once induced, ChiA is expressed in the main portion of the bacterial population and is actively secreted by a T10SS of *S*. Typhimurium.

## Results

### Chitinase A (ChiA) promotes adhesion and invasion of polarized intestinal epithelial cells

**Fig 1:**
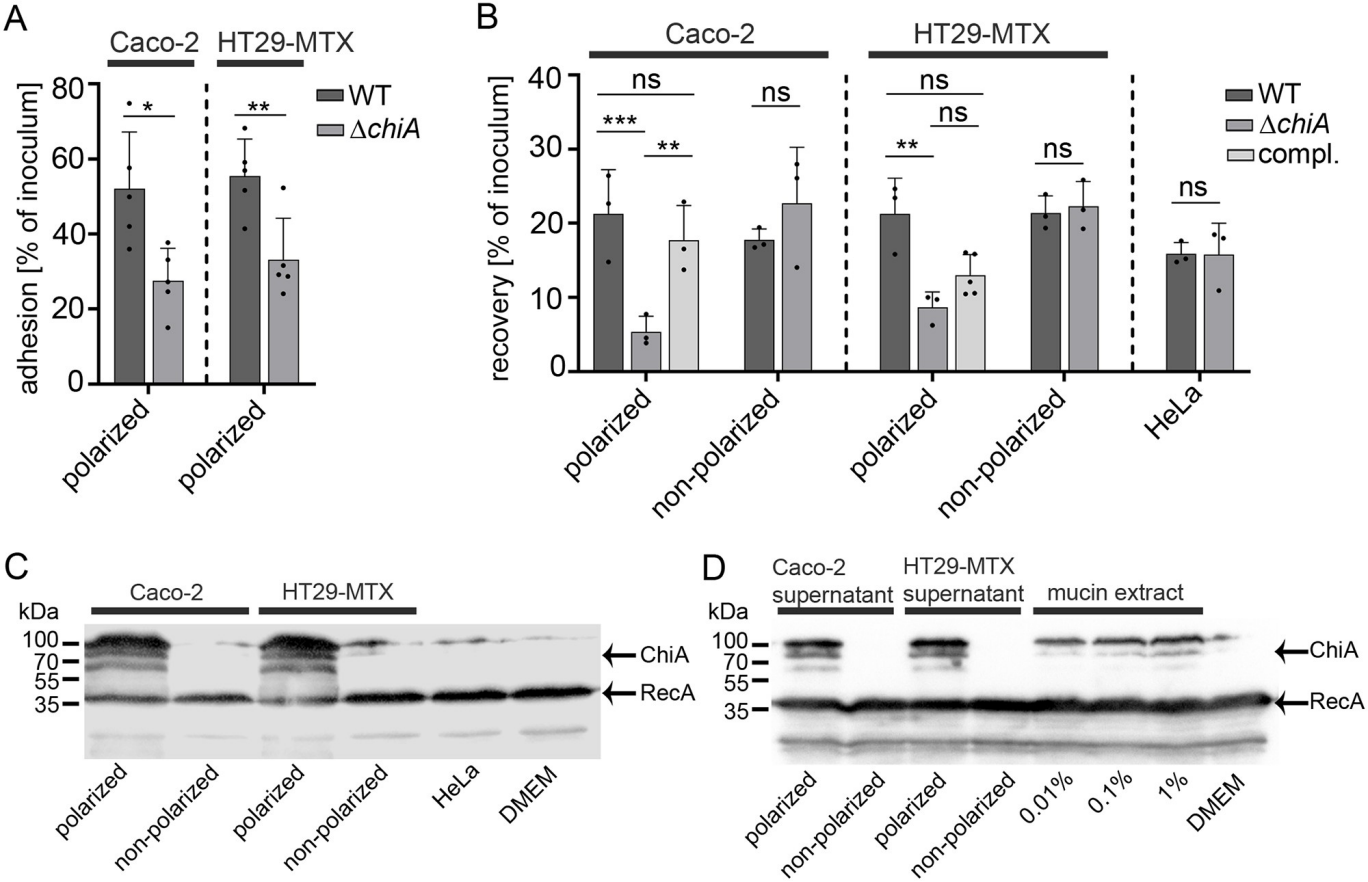
*S*. Typhimurium ChiA contributes to polarized IEC adhesion/invasion and is strongly induced upon contact with polarized IEC, polarized supernatants and mucin extracts. (A) Adhesion assay with *S*. Typhimurium wild-type and Δ*chiA* mutant in contact with polarized Caco-2 and HT29-MTX cells. Statistical analysis of adhesion versus inoculum (ratio) between conditions with unpaired *student´s* t-test. P>0.05: ns; P≤0.05: *; P≤0.01: **. (B) Recovery counts of *S*. Typhimurium WT, Δ*chiA* mutant, and complemented Δ*chiA* mutant strain after invasion assay (gentamycin treatment) in polarized and non-polarized Caco-2 cells, HT29-MTX cells, and HeLa cells. Counts of invaded bacteria were compared to inoculum (ratio) for each condition. Statistical analysis was implemented with ordinary one-way ANOVA and Bonferroni´s multiple comparison test P>0.05: ns; P≤0.05: *. (C) Expression analysis by Western blot of *S*. Typhimurium, carrying chromosomally encoded 3xFLAG tagged ChiA and plasmid pTG0069 (RecA-3xFLAG), after 3 h in contact with polarized and non-polarized Caco-2, HT29-MTX cells, and HeLa cells. The protein mass of ChiA is 79.3 kDa which also includes the C-terminal 3xFLAG tag. Hence, ChiA is arrow marked at about 80 kDa. The two additional protein bands at 60 and 100 kDa are unknown and indicate an aberrant migration behavior of ChiA on SDS-PAGE. RecA was used as a loading control. (D) The same strain as described before was used to detect ChiA expression after 3 h incubation of the bacteria in filtered (0.45 μm filter) cell-free supernatants of polarized and non-polarized IEC, or in a porcine stomach mucin extract (Sigma) of different concentrations as indicated in the figure.

Chitinases from various pathogenic bacteria have been shown to promote the binding of bacteria to host target cells followed by the invasion of the host cells. Therefore, we aimed to investigate whether chitinase A (ChiA) of *S*. Typhimurium has similar effects on *Salmonella*`s interaction with host target cells. We generated a clean Δ*chiA* deletion mutant in *S*. Typhimurium and carried-out cell adhesion assays. As host target cells, two different polarized human intestinal epithelial cells (IEC), absorptive Caco-2 enterocytes and mucus-secreting HT29-MTX enterocytes were used for the assays [28]. Before and during infection, the cells were treated with cytochalasin D to inhibit actin-driven uptake of the bacteria. After infection, the cells were rigorously washed and bacteria were plated on LB agar plates for CFU counts, which were calculated relative to the initial bacterial inoculum. Towards polarized Caco-2 and HT29-MTX cells, the Δ*chiA* mutant showed significantly reduced adherence of 25% of the inoculum compared to the wild-type showing about 55% adherence relatively to the inoculum (Fig 1A). After *Salmonella* adheres to its target cell it induces its invasion into the host cell. Following this infection pathway, wild-type *S*. Typhimurium and the corresponding Δ*chiA* mutant were used to infect polarized and non-polarized Caco-2 and HT29-MTX enterocytes. We were interested in how many intracellular bacteria can be recovered by realizing gentamycin protection assays. For cell polarization, cell confluency was maintained over 21 days before the co-incubation with *Salmonella*. After infection of the polarized host cells, gentamycin was added to the medium to kill residual bacteria in the cell culture medium. The protection assay clearly showed that the recovery rate of the Δ*chiA* mutant in both polarized IEC was significantly reduced compared to wild-type *S*. Typhimurium (Fig 1B). The diminished phenotype was fully complemented for Caco-2 cells, after introducing *chiA* on a plasmid in the *chiA* mutant strain, and partially complemented using the same complemented strain for HT29-MTX cells (Fig 1B). Interestingly, non-polarized IEC of both cell lines, as well as cervix epithelial cells (HeLa), showed no diminished recovery rates of the Δ*chiA* mutant compared to wild-type *S*. Typhimurium (Fig 1B). With these analyses, we showed that ChiA has a significant impact on *S*. Typhimurium infection efficiency of polarized human IEC.

### ChiA is efficiently expressed in contact with polarized human intestinal epithelial cells

Since ChiA-dependent invasion phenotypes could only be detected with polarized Caco-2 and HT29-MTX enterocytes, we examined whether this phenotype also correlates with a specific ChiA expression pattern. In a host cell-bacteria contact assay, *S*. Typhimurium, carrying chromosomally-encoded 3xFLAG-tagged ChiA, was incubated with polarized and non-polarized Caco-2 and HT29-MTX cells, as well as HeLa cells. After 3 h, the cell culture supernatants, which include the bacteria, were collected and the expression of ChiA was analyzed by Western blot analysis. In line with the invasion results, ChiA expression in *S*. Typhimurium was only detectable upon contact with polarized IEC (Fig 1C). In contrast, contact with non-polarized enterocytes as well as non-intestinal epithelial cells such as HeLa cells did not show detectable ChiA in the bacteria. Next, we were interested which intrinsic properties of polarized IEC are responsible for the detectable ChiA induction. Polarized IEC produce and release mucus in particular HT29-MTX cells that were used for the experiments. Thus, supernatants of polarized and non-polarized IEC were carefully taken and filtered through a 0.45 μm filter. *S*. Typhimurium, carrying chromosomally-encoded 3xFLAG-tagged ChiA, was incubated in these cell-free supernatants for 3 h and ChiA expression was analyzed by Western blot analysis. The ChiA expression patterns clearly showed that cell-free supernatants of polarized IEC induce ChiA expression (Fig 1D). Interestingly, cell-free supernatants taken from polarized Caco-2 cells induce ChiA expression indistinguishable from mucus-secreting HT29-MTX cells, albeit the amount of produced mucus is expected less in this cell type. We further examined whether mucins, the glycoprotein contents of cellular mucus, could be the responsible factor for ChiA induction. Here the same strain, carrying chromosomally-encoded 3xFLAG-tagged ChiA, was incubated in different concentrations of a commercially available partially purified mucin extract, isolated from the porcine stomach epithelium (Sigma). The Western blot analysis showed that the mucin extract induced ChiA expression, albeit less prominent than polarized cell-free supernatants (Fig 1D). Similar to cell-free supernatant results, the mucin extract induced *chiA* on the transcriptional level as shown by fluorescence promoter assays realized using a *chiA*::*sfGFP* reporter strain (S1 Fig). The Western blot analyses clearly demonstrate that only polarized IEC efficiently induce ChiA expression and that most probably the cellular mucus or mucin glycoproteins respectively are responsible for the induction, which subsequently causes ChiA-dependent increased adhesion and invasion into these cells.

### ChiA expression is stringently regulated by the positive transcriptional regulator ChiR

**Fig 2:**
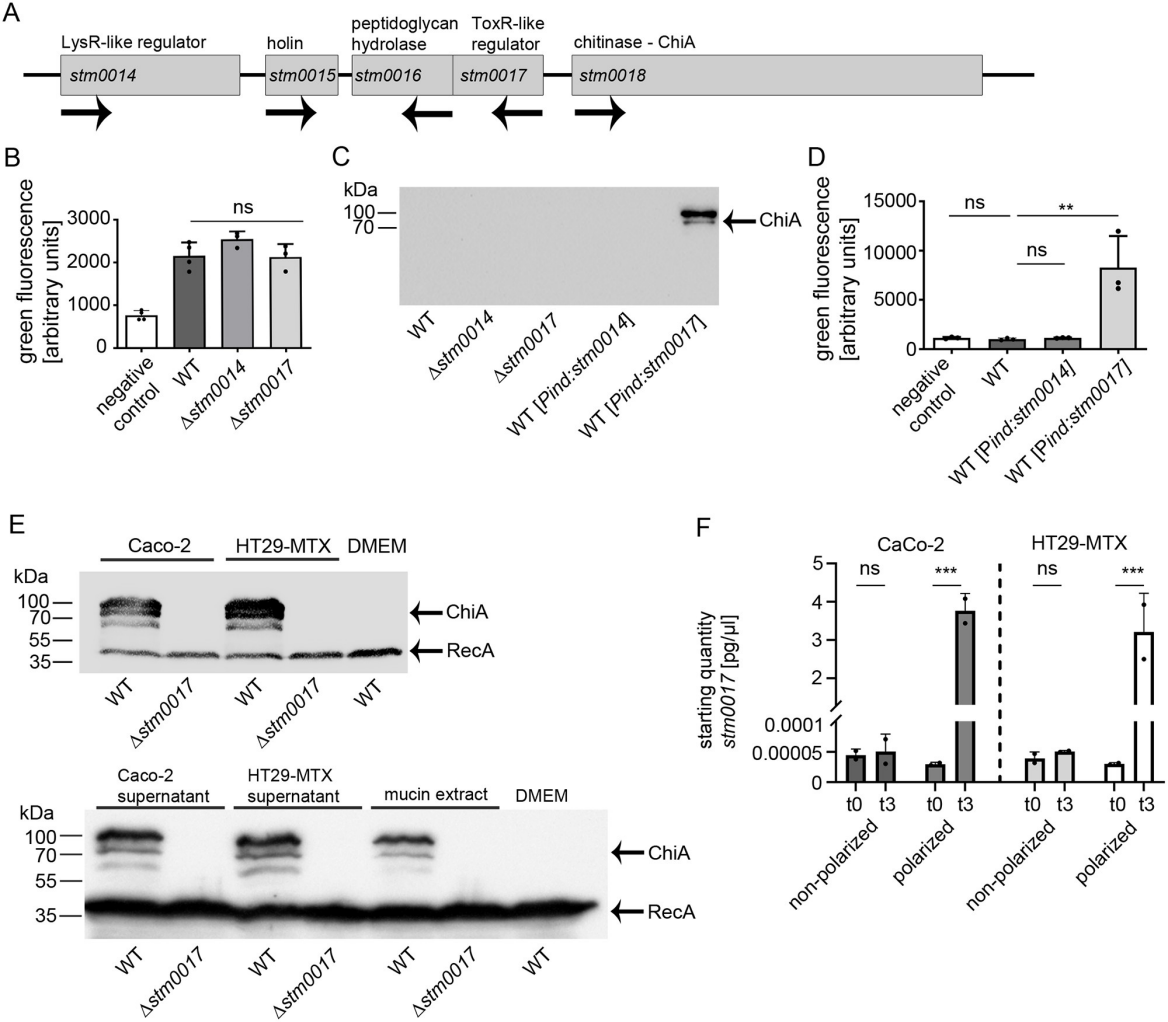
ChiA expression is strictly regulated by the chitinase regulator ChiR (STM0017). (A) Schematic overview of the chitinase A operon in *S*. Typhimurium. (B) *chiA* promoter activity assay of wild-type *S*. Typhimurium and clean deletion mutants Δ*stm0014* and Δ*stm0017* each carrying a plasmid expressing *sfGFP* under the control of the *chiA* promoter (*chiA-P_sfGFP*). As a negative control, *S*. Typhimurium wild-type carrying a promoterless_sfGFP plasmid was used. The strains were grown in standard LB rich medium for 6 h and harvested for fluorescence intensitity analyses. Statistical analysis was implemented with ordinary one-way ANOVA and Bonferroni´s multiple comparisons test P>0.05: ns. (C) Detection of ChiA expression by Western blot analysis in *S*. Typhimurium wild-type, Δ*stm0014*, and Δ*stm0017* mutant strains, carrying chromosomally encoded 3xFLAG tagged ChiA. To overexpress *stm0014* and *stm0017*, genes were cloned in a rhamnose inducible plasmid pTG0034 or pTG0035 respectively. Strains were grown for 6 h in standard LB rich medium. To induce the plasmids, 0.1% rhamnose was added to the medium for 3 h. (D) *chiA* promoter activity assay with S. Typhimurium carrying a chromosomal replacement of *chiA*::*sfGFP*. In this strain, plasmids pTG0034 (*stm0014*) or pTG0035 (*stm0017*) were introduced for overexpression analyses under LB rich medium growth conditions. Statistical analysis was implemented with ordinary one-way ANOVA and Bonferroni´s multiple comparisons test P>0.05: ns; P≤0.05: *; P≤0.01: **. (E) ChiA expression analysis by Western blot. Indicated *S*. Typhimurium strains (wild-type and Δ*stm0017*), carrying chromosomally encoded 3xFLAG tagged ChiA and plasmid pTG0069 (RecA-3xFLAG), were incubated for 3 h with polarized Caco-2 and HT29-MTX cells, cell-free supernatants of polarized Caco-2 and HT29-MTX cells, and 1% porcine mucin extract (Sigma) and harvested for Western blot analysis. RecA was used as a loading control. (F) Quantitative RT-PCR analysis of *stm0017* mRNA levels of *S*. Typhimurium before and after 3 h contact with polarized and non-polarized Caco-2 and HT29-MTX cells. Statistical analysis was implemented with unpaired *student`s* t-test P>0.05: ns; P≤0.001: ***.

In *Salmonella* Typhimurium, ChiA is encoded within a distinct operon, that consists of *chiA* (*stm0018*), a putative holin (*stm0015*), a putative peptidoglycan hydrolase (*stm0016*), and two putative regulators (*stm0014* and *stm0017*) (Fig 2A). In *Salmonella*, the regulation of *chiA* has not been examined yet. Before, studies on chitinases in *Serratia marcescens* revealed a similarly structured chitinase operon that contains a winged helix-turn-helix DNA-binding LysR-type transcriptional regulator, homologous to predicted STM0014 protein in *S*. Typhimurium [17,29]. To analyze the potential effects of transcriptional regulators STM0014 and STM0017 on the promoter activity of *chiA*, we generated a fluorescence reporter strain in which *chiA* was chromosomally replaced by sfGFP (*chiA*::*sfgfp*). Clean deletions of *stm0014* or *stm0017* were introduced and the resulting mutant strains were grown in LB medium and analyzed by fluorescence promoter activity assays. Both single deletions did not lead to an altered *chiA* promoter activity compared to the wild-type reporter strain (Fig 2B). Also, since none of the deletions led to an increased promoter activity of *chiA*, a repressor function can be ruled out for both regulators. The results were verified on the protein level by using a 3xFLAG-epitope tagged ChiA strain with corresponding regulator mutations (Fig 2C). Next, we artificially overexpressed both regulators from rhamnose-inducible plasmids. The induction of *stm0014* had no impact on the *chiA* promoter activity nor the expression of ChiA (Fig 2C and 2D). In contrast, overexpression of *stm0017* led to a strong promoter activity of *chiA* (Fig 2D), which we confirmed by the detection of high amounts of ChiA by Western blot analyses. (Fig 2C). To examine whether STM0017 also plays a role in the induced ChiA expression pattern in contact with polarized IEC, cell-bacteria contact assays as described before were realized. Wild-type and Δ*stm0017 S*. Typhimurium strains, carrying chromosomally-encoded 3xFLAG-tagged ChiA, were incubated with polarized Caco-2 or HT29-MTX cells and collected afterwards. ChiA protein expression was only detected in wild-type *S*. Typhimurium, showing that regulator STM0017 is crucial for induced ChiA expression in contact with the polarized IEC (Fig 2E). Quantitative RT-PCR analyses detected a strong increase of *stm0017* transcript amounts in bacteria collected from polarized cell contacts (Fig 2F). Here again, the polarization state of the cells was essential to increase *stm0017* transcripts, which as a consequence leads to the induced ChiA production upon polarized cell contact detected by the Western blot analyses. The results clearly show that STM0017 represents the major regulator for ChiA in *S*. Typhimurium and was therefore renamed ChiR. ChiR, by homology analyses, belongs to the family of ToxR-like transcriptional regulators and contains a C-terminal transmembrane domain (S2 Fig). Since *S*. Typhimurium harbors three additional ToxR-like transcriptional regulators, predicted to be encoded by *stm0029*, *stm0031*, *and stm3759*, we examined whether these regulators may also contribute to the induction of ChiA expression. The genes of *stm0029* and *stm0031* are located in close proximity to the chitinase operon. All three putative regulators contain one transmembrane domain similar to ChiR (S2 Fig) and their protein sequences show high similarities (STM0029 (76%), STM0031 (72%), and STM3759 (54%)) to ChiR. To test their potential impact on ChiA induction, we overexpressed the homologous regulators and analyzed ChiA expression by Western blot analysis. However, none of the homologues induced ChiA expression in contrast to ChiR (S2 Fig). Therefore, ChiR so far is the only known regulator in *S*. Typhimurium that specifically induces ChiA expression.

### In contact with polarized intestinal epithelial cells, ChiA is expressed in a major portion of the bacterial population

**Fig 3:**
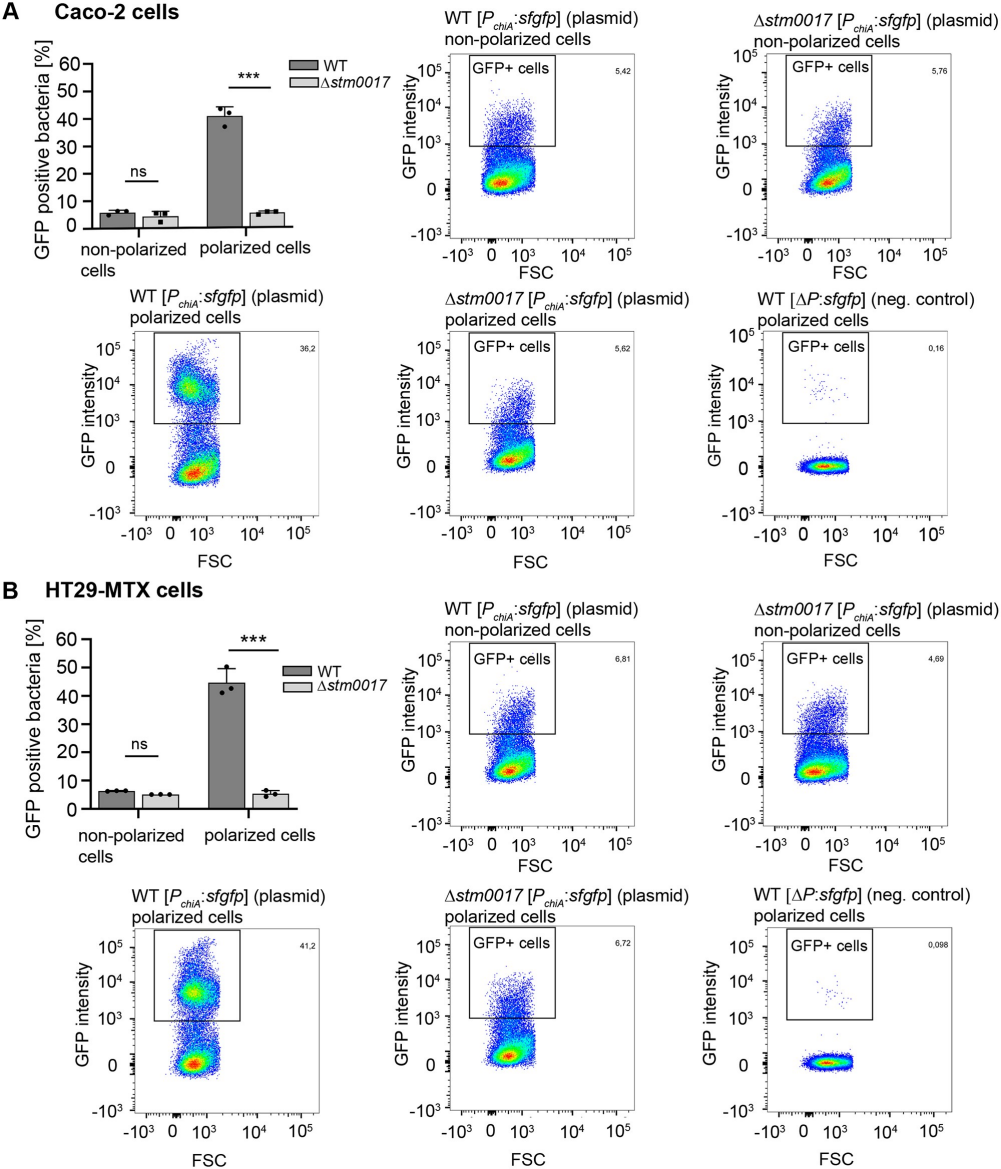
Quantification of *chiA* expressing *S*. Typhimurium subpopulations in contact with polarized intestinal epithelial cells. The indicated *S*. Typhimurium fluorescence reporter strains were incubated for 3 h in contact with polarized and non-polarized Caco-2 (A) or HT-29 MTX cells (B). The bacteria were harvested and analyzed by flow cytometry to quantitate the subpopulations of GFP-expressing bacteria. The dot plots identify the gate that were set to determine GFP positive (GFP+) bacterial cells. The non-GFP expressing S. Typhimurium strain WT Δ*P*:*sfgfp* in contact with polarized IEC was used to evaluate background fluorescence. The bar graphs show the average percentages ± standard deviation of GFP positive cells derived from three independent experiments. Statistical analyses were implemented with ordinary one-way ANOVA and Bonferroni´s multiple comparisons test P>0.05: ns; P≤0.05: *; P≤0.01: **; P≤0.001: ***.

The analyses of ChiA by Western blot analyses and fluorescence promoter assays revealed the induction of ChiA by polarized IEC on transcriptional and translational levels. However, these analyses do not provide detailed information on how many bacteria or what populations of bacteria respectively are responsible for the detected ChiA production. For *Serratia marcescens*, only a subpopulation of 1–14% of analyzed bacteria expresses chitinolytic proteins [17]. To address this question in *S*. Typhimurium, we used fluorescence reporter strains, in which *chiA* was chromosomally replaced by sfGFP (*chiA*::*sfgfp*) or by strains expressing plasmid-borne *chiA*_*promoter_sfGFP* constructs (*PchiA*:*sfgfp*). In cell contact assays, we collected the reporter strains after 3 h incubation with polarized and non-polarized IEC and measured the fluorescence intensities of the bacterial cells by flow cytometry. The plasmid-borne fluorescence reporter strain showed 40–50% GFP-positive bacteria in contact with polarized IEC and only about 5% GFP-positive bacteria in contact with non-polarized IEC (Fig 3A). This was confirmed by the chromosomal fluorescence reporter strain, which showed 47–53% GFP-positive bacteria in contact with polarized IEC (S3 Fig). For this strain, no GFP-positive bacteria were detected in contact with non-polarized IEC, comparable to the wild-type *S*. Typhimurium strain (S3 Fig). The important contribution of regulator ChiR to the GFP-positive bacterial subpopulation was verified by introducing a Δ*chiR* (Δ*stm0017*) mutation into the plasmid-borne fluorescence reporter strain. In contact with polarized IEC, this mutant strain showed a significantly diminished amount of 5% GFP-positive bacteria, similar to the contact with non-polarized IEC (Fig 3A). Overall, the cytometry analyses showed that in contact with polarized IEC, *chiA* is expressed in a major portion of the bacterial population and that this expression is driven by ChiR, the main regulator of ChiA in *S*. Typhimurium.

### A holin/peptidoglycan hydrolase-dependent protein secretion system actively secretes ChiA from *S*. Typhimurium

**Fig 4:**
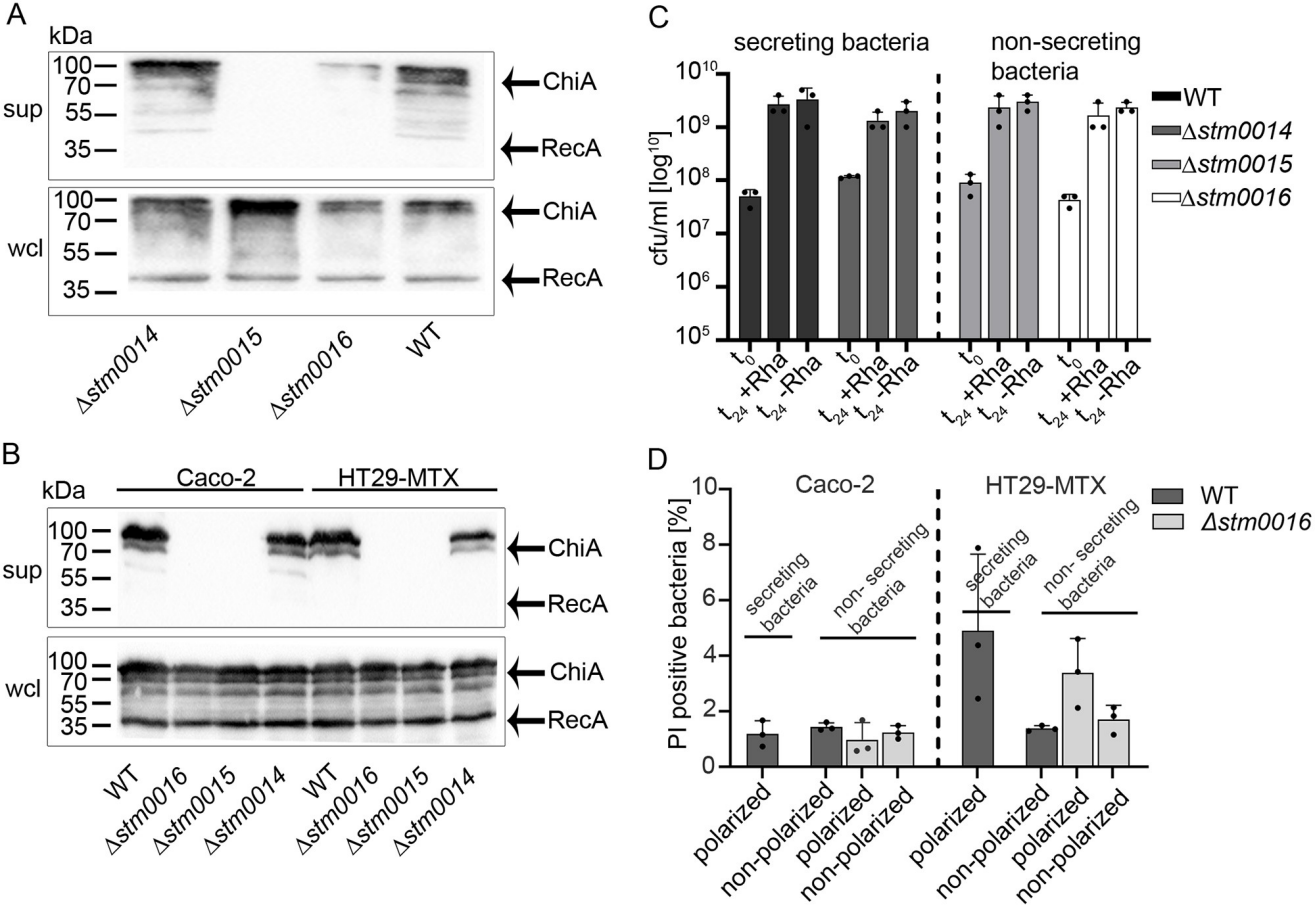
The secretion of ChiA depends on a holin and a peptidoglycan hydrolase. (A) The secretion of ChiA in bacterial cultures. *S*. Typhimurium wild-type, Δ*stm0014* (putative regulator), Δ*stm0015* (holin), and Δ*stm0016* (peptidoglycan hydrolase) mutants, carrying chromosomally encoded 3xFLAG tagged ChiA, a rhamnose inducible plasmid pTG0035 (*chiR/ stm0017*), and plasmid pTG0069 (RecA-3xFLAG) were used. The expression of ChiA was induced by adding 0.1% rhamnose to the chemical definded medium TTIM to overexpress *chiR* (*stm0017*). The Western blot analysis shows the expression of ChiA in the whole cell lysates (wcl) and its secretion to the cell-free bacterial supernatants (sup) after 24 h growth. (B) The secretion of ChiA upon contact with polarized IEC. The same set of strains as mentioned before was used to analyse ChiA secretion upon contact for 3 h with polarized Caco-2 and HT29-MTX cells. The Western blot analysis shows the expression of ChiA in the whole bacterial cell lysates (wcl) and its secretion in the cell-free bacterial supernatants (sup). The cytoplasmic RecA protein was used as a negative control for bacterial cell lysis. (C) CFU counts of indicated bacterial strains under bacterial culture conditions permissive for ChiA secretion as shown before (A). The wild-type and indicated mutant strains were harvested and plated on LB agar plates before (t_0_) and after 24 h growth with rhamnose (t_24_ + Rha) to induce ChiA expression and secretion and without rhamnose (t_24_—Rha), a condition that does not lead to ChiA expression and secretion. The CFU counts were quantitated, to demonstrate that CFUs of strains positive for ChiA secretion (wild-type and Δ*stm0014*) were indistinguishable to strains impaired in ChiA secretion (Δ*stm0015* and Δ*stm0016*). In addition, the induction of ChiA by *stm0017* overexpression (+ rhamnose) did not result in CFU differences when compared to non-ChiA-inducing conditions (- rhamnose). (D) Propidium iodide (PI) staining of bacteria in contact with polarized and non-polarized IEC. After 3 h contact with indicated IEC, bacteria were stained with PI and analyzed by flow cytometry. Displayed is the proportion (percentage) of PI-positive bacteria (dead bacteria) compared to the total pool of analyzed bacteria. Statistical analyses were implemented with ordinary one-way ANOVA and Bonferroni´s multiple comparisons test with a P value of 0.6074 (Caco-2) and 0.0741 (HT29-MTX), and a P value summary of not significant (P>0.05).

With *stm0015* and *stm0016*, two additional genes are located within the *Salmonella* chitinase operon (Fig 2A). Whereby *stm0015* is assumed to encode for a putative holin, a small pore-forming protein with a transmembrane domain, *stm0016* encodes for a putative peptidoglycan hydrolase predicted to be active on bacterial peptidoglycan due to its sequence homology to other peptidoglycan hydrolases. Protein sequence alignments of STM0016 show strong similarities to typhoid toxin secretion protein A (TtsA), a muramidase responsible for typhoid toxin secretion in *S*. Typhi [12,13]. In general, holins and peptidoglycan hydrolases in close proximity to toxins or large extracellular enzymes have been described as components of a holin/peptidoglycan hydrolase-dependent protein secretion system or Type 10 Secretion System (T10SS), which is responsible for the secretion of such cargo proteins in various bacterial pathogens [13–15]. Therefore, we hypothesized that this secretion system might also be responsible for the secretion of ChiA in *S*. Typhimurium. To test this hypothesis, we cultured wild-type *S*. Typhimurium with chromosomally-encoded 3xFLAG-tagged ChiA in vitro and analyzed cell-free supernatant contents for ChiA. For the induction of ChiA expression under in vitro bacterial culture conditions, we induced the transcriptional regulator ChiR encoded on a rhamnose-inducible plasmid. By Western blot analyses, we detected ChiA in the bacterial supernatants, providing the first evidence that ChiA, once expressed by *S*. Typhimurium, is efficiently secreted into the extracellular milieu (Fig 4A). In contrast, clean deletion mutants in *stm0015* (encoding for the putative holin) or *stm0016* (encoding for the putative peptidoglycan hydrolase) did not secrete ChiA into the bacterial supernatants even though induced ChiA expression levels were indistinguishable from the wild-type strain (Fig 4A). Therefore, we concluded that the holin and the peptidoglycan hydrolase are essential components for ChiA secretion. To investigate whether the pore-forming holin or the cell wall active peptidoglycan hydrolase leads to bacterial lysis, 3xFLAG tagged-RecA encoded on a plasmid was introduced in the strains tested. RecA, a mere cytoplasmic protein, was not detected in any of the bacterial supernatants, but most notably, was absent in the supernatant of ChiA secreting wild-type *S*. Typhimurium (Fig 4A). Thus, bacterial cell lysis, accompanied by an unspecific release of cytoplasmic contents was excluded. Of note, the deletion of *stm0014*, encoding for a putative LysR-type regulator, did not affect the secretion of ChiA.

Next, we were interested whether a condition that naturally induces the expression of ChiA also leads to ChiA secretion. Therefore, we performed cell contact assays, shown to induce ChiA expression (Fig 1D), with the same strains used for the in vitro culture supernatant experiments. The results confirmed the in vitro data, such that only wild-type *S*. Typhimurium and the Δ*stm0014* mutant in contact with polarized IEC, were able to secrete ChiA, whereas deletions of *stm0015* or *stm0016* completely abolished the secretion (Fig 4B). Again, the cytoplasmic control protein RecA was not detected in any of the strain supernatants (Fig 4B). In addition, CFU counts of *S*. Typhimurium grown under in vitro growth conditions permissive for ChiA secretion clearly indicated that during the secretion of ChiA, no decrease of viable bacteria can be detected (Fig 4C). On the contrary, ChiA secreting wild-type bacteria numbers increased during the secretion process, comparable to bacteria numbers of secretion deficient mutants. A direct measurement of bacterial cell death (loss of membrane integrity) by propidium iodide (PI) staining and flow cytometry showed that the percentage of PI positive bacteria remains low at 2–5%, and no significant differences in cell death between secreting and non-secreting bacteria were detected (Fig 4D and S4 Fig). Therefore, all data suggest a distinct and active secretion mechanism for ChiA, facilitated by the holin STM0015 and the peptidoglycan hydrolase STM0016, without a lytic loss of the secreting bacteria.

**Fig 5:**
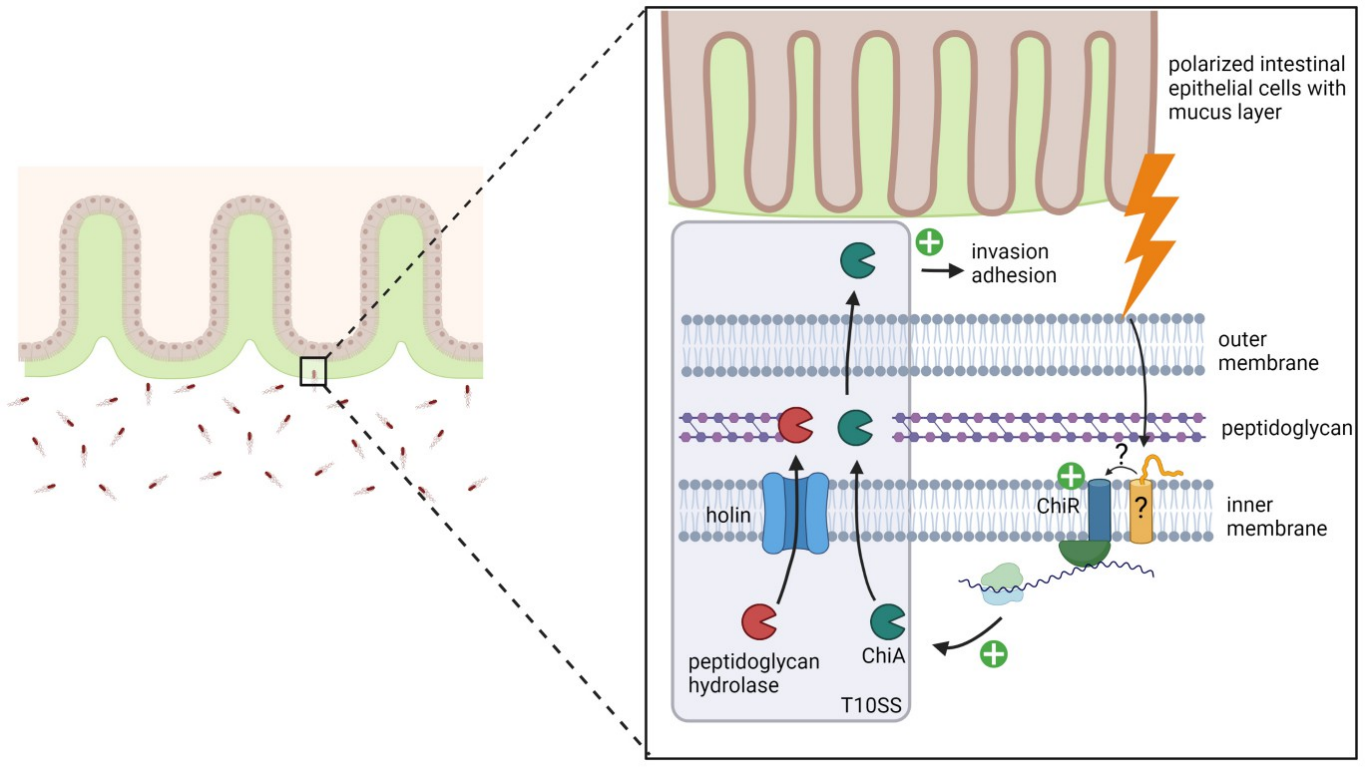
Proposed model for the controlled expression and secretion of virulence factor ChiA in S. Typhimurium. Upon contact with polarized IEC, mucin glycoproteins of the mucus layer induce the expression of the inner membrane-bound transcriptional regulator ChiR. ChiR accumulation subsequently leads to the induction of ChiA expression. Once ChiA is expressed, it is efficiently secreted by a holin/peptidoglycan hydrolase-dependent protein secretion system, also referred to as the Type 10 Secretion System (T10SS). Secreted ChiA then promotes the adherence of *S*. Typhimurium to intestinal epithelial cells, followed by increased invasion into these host cells. The model was created using biorender.com.

In summary, our study shows that upon contact with polarized IEC, the transcriptional regulator ChiR is strongly induced which leads to the induction of ChiA expression. Subsequently, a Type 10 Secretion System (T10SS) actively secrets ChiA to the extracellular environment, in which ChiA promotes the adherence of S. Typhimurium to intestinal epithelial cells, followed by increased invasion of these host cells (Fig 5).

## Discussion

*Salmonella enterica* serovar Typhimurium is a facultative intracellular pathogen and is considered as a prototypical broad-host-range serovar. This "generalist" is frequently associated with disease in humans, livestock, fowl, rodents, and birds, but has also been isolated from reptiles such as tortoises [30,31]. Its highly sophisticated ability to adapt to various hosts is reflected by a plethora of virulence factors that help to facilitate infection under various environmental conditions [32]. Besides classical virulence factors such as toxins, adhesins, and flagella, recent studies show that metabolic proteins such as host nutrient-degrading enzymes also play an important role in the establishment of *Salmonella* infection [33]. Pointing in that direction, a functional chitinase, able to degrade chitin, has been discovered in *Salmonella* Typhimurium [34]. Chitin, a linear polymer of β-1, 4-N-acetylglucosamine is the second most abundant biopolymer on the planet and can be found in fungi, algae, insects, crustaceans, and internal structures of invertebrates [35]. For marine bacteria such as *Vibrio* spp, which infects various chitinous hosts, the presence of functional chitinases is unquestioningly useful. They are involved in nutrient acquisition and promote survival in the marine environment [36]. In contrast, despite its broad host range, *S*. Typhimurium has only been isolated from chitin-free hosts. Even more striking is the discovery of an identical chitinase in the closely related *Salmonella* Typhi serovar, which represents a strictly human-adapted pathogen [12]. Therefore, *Salmonella* chitinases must have alternative functions beyond chitin hydrolysis to promote *Salmonella* infection. First hints for alternative targets provided studies from Leisner and colleagues which reported that *S*. Typhimurium ChiA can hydrolyze N-acetyllactosamine (LacNAc) motifs that terminate glycoproteins and glycolipids on mammalian cells as well as LacdiNAc motifs that can be found in the mammalian glycome [34,37]. In addition, glycan array scans to screen for ligand binding by ChiA revealed binding affinities to LacNAc structures of glycans [37]. Transcriptional profile analyses of intracellular S. *Ty*phimurium in epithelial and macrophage-like host cells, as well as in the cecum of chicken, demonstrated the upregulation of *chiA* [38–41]. In line with these results of host cell-driven *chiA* inductions and potential targets on host cells, we found that the mutation of *chiA* leads to the diminished attachment of *S*. Typhimurium to human IEC and subsequently to less invasion (Fig 1A and 1B). Our results provide strongly increased evidence towards two very recent studies of *S*. Typhimurium and *S*. Typhi, which demonstrated that ChiA has an influence on virulence, promoting adhesion to human epithelial cells, followed by increased invasion [26,27]. However, those studies did not explore ChiA expression, regulation, and secretion phenotypes, dependent on polarized cells. Interestingly, our study, in conformation of the other previous studies, also offers a plausible explanation for the finding that the deletion of all three known invasion factors (T3SS-1, RcK, and PagN) of *S*. Typhimurium did not completely abolish the invasion in host cells [42]. We surmise that ChiA is the missing candidate that partially compensates for the loss of the other invasion factors. Another factor that contributes to *S*. Typhimurium`s ability to adhere and to invade host cells are flagella [43–45]. Nevertheless, under conditions of ChiA mediated cell adherence and invasion the expression of flagellin FliC remained unchanged and was indistinguishable from cell-free medium controls (S5 Fig). In addition, neither the deletion of gene *stm0017*, that encodes for ChiR the positive regulator of ChiA, nor its overexpression leading to high ChiA expression had any detectable effects on FliC expression. Therefore, we conclude that ChiA mediated cell adherence and invasion has no impact on flagella production in *S*. Typhimurium and is not correlated to flagella-mediated cell adherence and invasion.

Intriguingly, in our study, the adherence and invasion deficiency of the Δ*chiA* mutant and the induction of ChiA strictly depended on the polarization state of the IEC (Fig 1A–1C). As consequence thereof, non-polarized cells did not promote a ChiA-dependent adhesion or invasion phenotype. These findings are similar to results obtained in a previous study, using non-polarized human colonic T84 cells, where a single *chiA* mutation did not show significant reduction of adhesion and invasion of *S*. Typhimurium [26]. Many physiological, physical, and structural changes emerge during the process of epithelial cell polarization. The polarized monolayer is accompanied by the formation of apical filament-based microvilli and lateral tight junctions, apical specific membrane proteins such as apical brush-border proteins, and several pattern-recognition receptors [46]. Changes also include the localized, apical production of a mucus layer with underlying glycoproteins referred to as mucins. Interestingly, cell-free supernatants of polarized IEC, that contain secreted mucus, induced ChiA expression similar to direct contact of *S*. Typhimurium with polarized IEC (Fig 1D). Since mucus mainly consists of mucin glycoproteins these results are in line with the detected ChiA induction by a partially purified mucin extract. Both results indicate that mucin glycoproteins are potentially responsible for ChiA induction and therefore could be targets for chitinase A of *S*. Typhimurium. Attention should be paid to the fact that supernatants of mucus non-secreting Caco-2 cells also induced ChiA expression. Whether these supernatants also contain mucus, probably in lower amount but still enough to induce ChiA, needs to be investigated in future studies. Also, cell-free supernatants and the partially purified mucin extract from Sigma still contain several other unknown proteins and carbohydrates that could be responsible for the induction of ChiA [47]. In general, any of the apical cell changes could be targets for ChiA and thus decisive factors for ChiA-dependent adhesion/invasion phenotypes and will be investigated more comprehensively in future studies. However, differences in the strength and thickness of the mucus layer can be ruled out in our study, since strong mucus-producing and secreting HT29-MTX cells showed no differences in the adherence and invasion of the Δ*chiA* mutant compared to non-secreting low mucus-producing Caco-2 cells (Fig 1A and 1B). Of note, albeit not secreting mucus, Caco-2 cells sufficiently express mucin glycoproteins in their polarized state that might be targeted by ChiA [48].

ChiA protein expression under standard in vitro growth conditions such as LB rich medium was not detected by Western blot analysis, despite harvesting large amounts of bacteria (Fig 2C). This is in line with a low promoter activity of *chiA* detected by promoter fluorescence assays (Fig 2B). Similar results have been obtained by a quantitative study of *chiA* transcripts from *S*. Typhimurium grown in LB rich medium [26]. Thus, *chiA* expression seems to be tightly regulated, keeping its expression repressed under non-inducing conditions such as growth in vitro or in contact with non-polarized IEC. Upon receiving an inducing signal such as the contact with polarized IEC, *chiA* expression on mRNA and protein levels increases significantly (Figs 1C and 2D and S1 Fig). This induction is strictly mediated by the transcriptional regulator ChiR (STM0017), which is functionally characterized here for the first time (Fig 2C and 2E). The induced expression of *chiR* per se is sufficient for the subsequent induction of ChiA. In line with this, qRT-PCR results show that contact to polarized IEC strongly upregulated *chiR* transcript quantities (Fig 2F). A similar tight and direct regulation of chitinases has been reported for *Serratia marcescens* [17]. Here, a cytoplasmic Lys-R type transcriptional regulator (LTTR) directly induces the expression of otherwise poorly expressed chitinases. Interestingly, with STM0014, a homologous LysR-type regulator is also located in the *Salmonella* chitinase operon (Fig 2A). Nevertheless, neither deletion nor overexpression of *stm0014* had a marked impact on ChiA expression in *S*. Typhimurium under the conditions tested (Fig 2B and 2D), which is in stark contrast to chitinase regulation in *Serratia marcescens* [17]. In contrast to the likely cytoplasmic STM0014, ChiR shows domain homologies to the CadC transcriptional regulator that belongs to the family of inner membrane-bound ToxR-like activators [49–51]. These ToxR-like regulators are one-component systems that exhibit sensory, signal transduction, and effector function combined within one protein [51]. ChiR also consists of a single transmembrane domain and a cytoplasmic DNA-binding domain with a winged helix-turn-helix motif (S2 Fig). Interestingly, a periplasmic sensor domain is presumably missing, leaving the question open of how the inducing signal from polarized IEC can be sensed. Therefore, we hypothesize that at least one additional protein must interplay with ChiR and function as a co-sensor, similar to the CadC regulator system in *E*. *coli* [52]. Here, under non-inducing conditions, CadC is inhibited by the contact with a second protein LysP [53]. Upon induction, the CadC/LysP interaction gets destabilized, thereby permitting signal transduction towards CadC induction. One can assume that a similar mechanism could play a role in ChiA regulation, by inhibiting signal transduction towards ChiR under non-inducing conditions by an unknown protein partner.

In contact with polarized IEC, a *S*. Typhimurium population of about 50% showed *chiA* expression (Fig 3A and 3B, and S3 Fig). Similar results were obtained in *Serratia marcescens*, where overexpression of the chitinase regulator shifted the proportion of the cell population expressing chitinase genes from 1–14% to more than 60% [17]. Under conditions of ChiA expression, whether by the artificial induction of ChiR using a rhamnose-inducible plasmid or in contact with polarized IEC, ChiA was detected in the bacterial supernatants (Fig 4A and 4B). The accumulation of ChiA in the bacterial supernatants depended on the presence of gene *stm0015*, which encodes for a putative inner membrane pore-forming holin, and on *stm0016*, which encodes for a putative cell-wall active peptidoglycan hydrolase. Since both proteins are active against the bacterial cell wall, one can assume that their activities lyse the bacteria and subsequently release ChiA to the supernatants. However, we found that RecA, a cytoplasmic protein, was not detected in the bacterial supernatants (Fig 4A and 4B). By using this cytoplasmic control protein, bacterial cell lysis and hence passive and non-specific release of ChiA can be excluded. In line with this assumption are results of CFU counts, which unambiguously show that bacteria under conditions of ChiA secretion indicate no decline of viabilities, but on the contrary even show an increase in bacterial numbers (Fig 4C). Of note, since about 50% of the bacterial population express and presumably secrete ChiA, lysis should result in a severe drop of viable bacteria. But only a minor bacterial fraction of 2–5% showed positive PI staining indicating cell death, which is in a range that can be commonly detected by bacteria growing in standard bacterial cultures [54]. Some minor fluctuations occurred in contact with HT29-MTX cells, but no significant differences between secreting and non-secreting bacteria were noted, neither upon contact with IEC (Fig 4D) nor within bacterial cultures conditions (S4 Fig) that would correlate to the major portion (50%) of the bacterial population that expresses and secretes ChiA. Therefore, we suggest the active and non-lytic secretion of ChiA from *S*. Typhimurium by the combined actions of the holin STM0015 and the peptidoglycan hydrolase STM0016. Holins, peptidoglycan hydrolases, and cargo proteins in close proximity are characteristic components of a novel protein secretion system named holin/peptidoglycan hydrolase-dependent protein secretion system or T10SS [14]. In *S*. Typhi, such a secretion system has been investigated comprehensively and is responsible for the active secretion of typhoid toxin [12,13,55]. In *Serratia marcescens*, comprehensive studies have shown the active secretion of chitinases by the same secretion system [15]. Non-lytic holin/endolysin-dependent secretion mechanisms have also been reported for Gram-positive bacteria such as *Clostridium perfringens* and *Clostridioides difficile* [56,57]. With our study on chitinase A in *S*. Typhimurium, we have broadened the proven range of bacteria and virulence factors actively transported through this novel secretion mode of the T10SS.

## Material and methods

### Cloning/mutagenesis

The bacterial strains and plasmids used in this study are listed in S1 Table. All *Salmonella* Typhimurium strains are derived from *Salmonella enterica* Serovar Typhimurium SL1344 [58]. All in-frame deletions or insertions (3xFLAG epitope tag and sfGFP) into the *S*. Typhimurium chromosome were generated by standard recombinant DNA and allelic exchange procedures using, *E*. *coli* CC118(*λpir*) for plasmid amplification [59], *E*. *coli* β-2163 Δ*nic35* as the conjugative donor strain [60] and the R6K-derived suicide vector pSB890 as previously described [61]. Primers used for cloning procedures are listed in S2 Table. For *S*. Typhimurium *ΔchiA* complementation studies, we used plasmid pTG0073, which encodes a rhamnose-inducible promoter and is derived from plasmid pT12 [62]. sfGFP of indicated fluorescence reporter strains is obtained from plasmid pWSK167 [63]. All plasmids used in this study were constructed using the Gibson assembly cloning strategy, except plasmid pTG0073 which was digested by XhoI and EcoRI and ligated via a T4 ligase protocol [64]. All generated plasmids and strains used in this study have been verified by nucleotide sequencing.

### Bacterial culture

*S*. Typhimurium strains were routinely cultured on standard LB agar plates or in liquid LB broth (10 g/l NaCl, 10 g/l tryptone, 5 g/l yeast extract) on a shaking platform at 37°C. For invasion assays, adhesion assays and contact assays bacteria were subcultured (1:30) from overnight cultures in LB 0.3 M NaCl to induce SPI1-T3SS activity [65]. Subcultures were incubated until reaching an OD_600_ of 0.9 on a shaking platform at 37°C. For the in vitro bacterial culture, to test secretion of ChiA, the chemically defined medium TTIM was used as previously described [13]. When appropriate, antibiotics were added to bacterial cultures (5 μg/ml tetracycline (Sigma), 50 μg/ml kanamycin (Roth), 100 μg/ml ampicillin (Roth), or 30 μg/ml chloramphenicol (Roth)).

### Eukaryotic cell culture

The Caco-2 and HT29-MTX [66,67] cells were grown in Dulbecco’s modified eagle’s medium (DMEM, high glucose, with glutamine, Gibco), supplemented with 10% fetal calf serum (FCS, Gibco) and 1% non-essential amino acids (Gibco). For Caco-2 cells 1 mM sodium pyruvate (Gibco) was added to the medium. Penicillin/streptomycin (1%, Gibco) was used for long-term cultivation. Caco-2 and HT29-MTX cells were incubated for 21 days prior to be used as “polarized” cells [68] or incubated for one day prior to be used as “non-polarized” cells. Days were counted, from the time point when the cells showed confluence, and medium was changed every 2–3 days. The HeLa cells were grown in DMEM (high glucose, with glutamine), supplemented with 10% FCS. All cells were incubated at 37°C in a humidified incubator with 5% CO_2_.

### Gentamycin protection assay

Cells were seeded in 24-well plates and incubated for the appropriate number of days. For the host cell infection, the different *S*. Typhimurium strains were grown overnight and then sub-cultured (1:30) in fresh LB medium containing 0.3 M NaCl, to stimulate expression of the SPI-1 type III secretion system [65]. When the bacteria reached an OD_600_ of 0.9, the bacteria were diluted in DMEM to provide the desired bacterial amounts and added to the host cells. The cells were infected with 2x10^6^ bacteria/well for 60 minutes. Thereafter, the cells were washed three times with DPBS (Gibco) and further incubated for 60 min with DMEM supplemented with gentamicin (100 μg/ml) to kill remaining extracellular bacteria. Afterwards, the cells were lysed with 500 μl of 1% Triton-X100. After 10 min on a shaking platform, the cells were pipetted up and down to induce lysis. The wells were rinsed with 500 μl PBS and combined with the lysates. Dilution series of the lysates and the initial bacteria inocula were plated on LB agar plates to determine CFUs. The corresponding counts of invaded intracellular bacteria are displayed as percentage (ratio) to the corresponding total inocula per well.

### Bacterial adhesion assay to cells

Cells were seeded in 24-well plates and incubated for the appropriate number of days. Before the addition of the bacteria, the cells were treated with cytochalasin D (2 μg/ml) (Cayman chemical) for 1 h to inhibit actin-driven uptake of the bacteria as shown previously [69]. For testing the adhesion, the different *S*. Typhimurium strains were grown overnight and then sub-cultured (1:30) in standard fresh LB medium. When the bacteria reached an OD_600_ of 0.9, the bacteria were diluted in DMEM and added to the human cells in presence of cytochalasin D. Cells were infected with 4x10^5^ bacteria/well for 60 minutes. Thereafter, cells were rigorously washed five times with PBS to remove non-adherent bacteria. Afterwards, the cells were incubated with 500 μl 1% Triton-X100. After 10 min on a shaking platform, cells and adherent bacteria were collected. The wells were rinsed with 500 μl PBS and combined with the cell/bacteria samples. Dilution series of the adherent bacteria and the initial bacteria inocula were plated on LB agar plates to determine CFUs. The corresponding adhesion counts are displayed as percentage to the corresponding inocula.

### Host cell/bacteria contact assay, host cell supernatant and mucin extract assay, to determine ChiA expression and regulation

The cells were seeded in 6- or 12-well plates and incubated for the appropriate number of days. For the contact assay the cell culture medium DMEM was diluted 1:1 with PBS to limit the amount of glucose in the medium. The different *S*. Typhimurium strains were grown overnight and then sub-cultured (1:30) in fresh LB containing 0.3 M NaCl, to stimulate expression of the SPI-1 Type 3 Secretion System [65]. When the bacteria reached an OD_600_ of 0.9, they were diluted in DMEM and added to the fully confluent host cells. 2x10^8^ bacteria were added per well (for 6-well plates), or 7.3x10^7^ bacteria per well (for 12 well-plates), respectively. For host cell supernatant experiments, supernatants were carefully collected from polarized and non-polarized Caco-2 and HT29-MTX cells and filtered through a 0.45 μm filter (Millipore) to obtain cell-free supernatants. For the mucin extract assay, a stock (10%) of porcine stomach mucin extracts (Sigma) was resolved in DMEM medium and various concentrations (0.01%, 0.1%, and 1%) were applied in the experiments. The cells, cell-free supernatants, and mucin extracts were incubated together with the bacteria for 3 h. Bacteria directly (cell-free supernatants, mucin extracts) and bacteria from the cell culture supernatants (host cell/bacteria contact assay), were cautiously collected. For various analyses, the bacteria were further processed accordingly.

### Secretion assay for ChiA

To detect ChiA secretion in bacterial cultures, *S*. Typhimurium wild-type, Δ*stm0014*, Δ*stm0015*, and Δ*stm0016* deletion strains carrying chromosomally encoded 3xFLAG-epitope-tagged ChiA, were grown overnight in LB medium, washed twice with 1 x PBS and sub-cultured (1:50) in the chemically defined medium TTIM supplemented with 0.001% rhamnose [13]. Rhamnose was used to induce ChiA expression under in vitro bacterial culture conditions. Here, the tested strains carry a rhamnose-inducible plasmid pTG0035 that expresses *stm0017*. The strains also carry a second rhamnose-inducible plasmid pTG0069 (incl. a suitable origin of replication), that expresses 3xFLAG epitope-tagged RecA. The cytoplasmic localized RecA served as a negative control for cell lysis in the Western blot analyses of the bacterial supernatants and as a loading control for the whole cell lysates.

To detect ChiA secretion in contact with polarized IEC, *S*. Typhimurium wild-type and mutant strains in contact with polarized IEC cells (see host cell/bacteria contact assay), were collected. These strains also carry plasmid pTG0068 that expresses 3xFLAG epitope tagged RecA as a control protein as described above. The bacteria samples were centrifuged and bacteria pellets were resuspended in SDS Laemmli sample buffer (whole cell lysates). The supernatants were filtered (0.22 μm) and 10% trichloroacetic acid (TCA) was added for protein precipitation. The samples were incubated at 4°C overnight. After centrifugation (15,000 x g, 45 min), the supernatants were discarded, and pellets were washed twice with acetone followed by drying at room temperature. The pellets were resuspended in Laemmli SDS sample buffer. The presence of ChiA and RecA in the supernatants and in the bacterial pellets were determined by Western blot analyses.

### Western blot analyses

Samples for Western blot analyses were diluted in Laemmli SDS sample buffer, boiled for 5 min at 99°C and applied on a SDS gel (12%). The gel was blotted on 0.45 μm nitrocellulose blotting membrane (Amersham Protran 0.45 μm NC) for 1 h (20 V, 0.3 A). The membrane was blocked with TBS + 5% non-fat milk (Roth) for 30 min. The primary anti-FLAG M2 mouse monoclonal antibody (Sigma) was diluted 1:10,000 in TBS-T with 5% non-fat milk and applied at 4°C overnight. After removal of the primary antibody by washing the membrane three times with TBS-T, the secondary antibody (goat anti-mouse-HRP, 1:10,000) in TBS-T 5% non-fat milk was added to the membrane and further incubated for 1 h at room temperature. The membrane then was washed three times with TBS-T to remove the secondary antibody. Immobilon Crescendo Western HRP substrate (Millipore) was added on the membrane for 1 min and chemiluminescence was detected on a ChemiDoc station.

### Fluorescence reporter assay

For the analyses of the *chiA* promoter activity, *S*. Typhimurium fluorescence reporter strains were generated. To investigate the impact of mutations of *stm0014* and *stm0017* on the *chiA* promoter activity, a 658 bp region upstream of *chiA* was cloned in front of sfGFP to generate plasmid pTG0033. To investigate the impact of the overexpression of *stm0014* and *stm0017*, the genes were cloned into a rhamnose inducible plasmid, generating plasmids pTG0034 and pTG0035 (S1 Table). The strains were grown overnight in LB medium with appropriate antibiotics. The bacteria were washed with PBS and a fresh subculture with a starting OD_600_ of 0.1 in fresh LB medium was generated. The strains harboring plasmids pTG0034 and pTG0035 were incubated with 0.1% rhamnose to induce gene expression as described before. After 6 h, the bacterial cultures were diluted to an OD_600_ of 0.1 with PBS. The fluorescence intensities were measured in 96 well plates with 100 μl aliquots of the bacterial dilutions by a Clariostar plate reader (BMG lab tech). The presented values are mean values of three independent biological replicates. For the induction of the *chiA* promoter in polarized and non-polarized supernatants, and in mucin extracts a fluorescence reporter strain with chromosomally encoded *chiA*::*sfGFP* was used (S1 Table). The strain was grown overnight in LB medium. The bacteria were washed with PBS and a fresh subculture with a starting OD_600_ of 0.1 in fresh LB medium was generated. After 2 h the bacteria were washed with PBS and diluted 1/10 in the various cell-free supernatants or mucin extracts solved in DMEM and incubated for 3 h. Afterwards the bacteria were pelleted and resuspended in PBS. The fluorescence intensities were measured in 96 well plates with 100 μl aliquots of the bacterial samples by a Clariostar plate reader (BMG lab tech). The fluorescence intensities were normalized to equal bacterial numbers of 1x10^8^ bacteria/ml in each sample. For this purpose, bacterial CFU counts of different samples and conditions were determined by plating dilution series on LB Agar plates. The presented values are mean values of three independent biological replicates.

### Propidium iodide (PI) staining

*S*. Typhimurium wild-type and mutant strain Δ*stm0016* (peptidoglycan hydrolase), both carrying chromosomally encoded 3xFLAG-epitope-tagged ChiA, and rhamnose inducible plasmid pTG0035 (*stm0017*) were incubated under bacterial culture conditions permissive for ChiA secretion. After cultivation for 24 h in medium TTIM supplemented with 0.001% rhamnose to induce ChiA expression and secretion, 100 μl of the bacterial culture were stained with 1 μl of 1 mg/ml propidium iodide solution. Bacteria were incubated with PI for 1 h in the dark. As a positive control, bacteria were killed by incubation for 20 min at 75°C. As negative control bacteria from a shaking subculture (1:30), incubated for 3 h at 37°C were used. Both were stained in the same way as the strains of interest. Thereafter, the fluorescence intensity was measured by flow cytometry. The same strains, but without rhamnose inducible plasmid pTG0035 (*stm0017/chiR*), were used to test for bacterial viabilities in contact with polarized IEC. Bacteria and IEC were cultured and incubated as described under section cell contact assay. The supernatants (2 ml), including the bacteria, were collected and concentrated by centrifugation (3 min, 13,000 x g). 200 μl of the bacterial/cell supernatant suspensions were incubated with 2 μl of 1 mg/ml propidium iodide solution for 1 h in the dark and fluorescence intensities were measured there after by flow cytometry.

### Flow cytometry analysis

To analyze subpopulations of bacteria expressing *chiA*, supernatants of cell contact assays that contain bacteria, expressing *sfGFP* under the promoter of *chiA* (*PchiA*:*sfGFP*) on plasmid pTG0033 or chromosomally *chiA*::*sfGFP* replacement mutants, were measured by flow cytometry. Therefore, bacteria in the cell culture supernatants were pelleted at 13,000 rpm for 5 min. The pellets were resuspended in 4% paraformaldehyde (PFA) and incubated for 20 min for fixation. After centrifugation (10,000 g for 5 min) the pellets were washed and resuspended in PBS. The fluorescence intensities were measured by flow cytometry (BD CantoII). Green fluorescence was excited at 488 nm. Gates were set using a promoterless sfGFP plasmid (pTG0044) or a non-fluorescent wild-type S. Typhimurium strain as fluorescence negative controls. Propidium iodide-stained bacteria were measured without fixation. PI was excited at 530 nm and Gates were set using positive and negative control. The data were analyzed by using the FlowJo software (FlowJo LLC.).

### Bacterial RNA isolation and (q) RT-PCR

For the quantitative RT-PCR analyses, bacteria were harvested from cell contact assays as described before. The isolation of bacterial RNA and synthesis of cDNA was realized according to a previously described procedure by Estibariz *et al*. [70]. Briefly, the bacteria containing supernatants were collected and centrifuged (16,000 x g for 2 min, RT). The pellets were shock-frozen in liquid nitrogen and stored at -80°C. Bacteria were lysed in a Fastprep bead-beater (MP Biomedicals Inc., Santa Ana, CA, USA) using MP-Fastprep24 bacteria lysing matrix B followed by bacterial lysis for 45 s at a power setting of 5,5. The bacterial RNA was isolated with QIAGEN RNeasy Mini kit according to the manufacturer`s protocol after the additional lysis step. Using photometric measurements (nanodrop), agarose gels and a tape station (Agilent, Tape Station 4200, RNA Nano Kit, Agilent), the RNA was quantified and quality-controlled accordingly to the described procedure [68]. DNA contamination was eliminated with DNase I (TURBO RNAse-free DNA removal kit; Ambion) according to the manufacturer’s protocol. The cDNA synthesis was performed using Superscript III RT (Invitrogen) for 2.5 h at 42°C using random primers. The cDNA was diluted in 30 μl H2O and 1 μl was used for qPCR. Equal amounts of total RNA (500 ng) were used for cDNA biosynthesis to equalize cDNA amounts. Adjusted volumes of cDNA were used for (q)RT-PCR; in addition, transcript amounts were quantitated according to an internal standard (bacterial *stm0017* rRNA), with defined standard concentrations to determine absolute transcript amounts (in pg/μl). Primer used for (q)RT-PCRs are listed in S3 Table.

### Statistical analyses

Data were plotted using GraphPad Prism 9 software. All data were acquired from at least three biological replicates. The mean values for recovery and adhesion rates were determined and compared. Data of flow cytometry and measurement of GFP intensity with the Clariostar plate reader were acquired from three biological replicates. Statistical significance was evaluated with ANOVA (three or more groups), followed by Bonferroni´s multiple comparison test or by unpaired student’s *t*-test (two groups). Significance is given as follows: ns: p>0.05, *: p<0.05, **: p<0.01. ***: p<0.001.

### Prediction of transmembrane domains

For prediction of transmembrane helices, the prediction tool TMHMM—2.0 was used (https://services.healthtech.dtu.dk/service.php?TMHMM-2.0). Proteins were searched in FASTA formats and the probabilities for transmembrane domains are given as plots.

### Homology searches

Homologies were determined using protein blastp searches and alignments (NIH National Library of Medicine, https://blast.ncbi.nlm.nih.gov/Blast.cgi?PAGE=Proteins.).

## Supporting information

S1 Fig *chiA* promoter activity assay upon contact with cell-free supernatants of polarized IEC and mucin glycoprotein extracts. *S*. Typhimurium fluorescence reporter strain, carrying a chromosomal replacement of *chiA*::*sfGFP*, was incubated for 3 h in filtered cell-free supernatants of polarized and non-polarized IEC, and in various concentrations of mucin extracts (Sigma) as indicated. As a negative control, the same strain was grown in DMEM. The fluorescence intensities were normalized to equal bacterial numbers of 1x10^8^ bacteria/ml. Therefore, bacterial CFU counts were determined by plating dilution series on LB Agar plates. Statistical analysis was implemented with ordinary one-way ANOVA and Bonferroni´s multiple comparisons test P≤0.001: ***.
(TIF)

S2 Fig Homologous proteins of ChiR (STM0017) in *S*. Typhimurium do not induce ChiA expression. By using the Basic Local Alignment Search Tool for proteins (BLASTp) of the NIH (National Library of Medicine), three homologous proteins of STM0017 (ChiR) were identified. STM0029 and STM0031 show 49% (STM0029) and 48% (STM0031) sequence identities, and have similarities of 76% and 72%, respectively (red square). For the prediction of transmembrane helices, prediction tool TMHMM-2.0 (https://services.healthtech.dtu.dk/service.php?TMHMM-2.0) was used. The proteins were searched in FASTA formats and the probabilities for transmembrane domains are indicated as plots. The three homologous regulators were overexpressed using rhamnose inducible plasmids in a *S*. Typhimurium strain that expresses chromosomally integrated 3xFlag-tagged ChiA. The Western blot analysis showed that none of the tested homologues of ChiR (STM0017) led to ChiA expression.
(TIF)

S3 Fig Quantification of *chiA* expressing *S*. Typhimurium subpopulations in contact with non-polarized and polarized intestinal epithelial cells. The indicated *S*. Typhimurium fluorescence reporter strains with chromosomal replacements *chiA*::*sfGFP* were incubated for 3 h in contact with polarized and non-polarized Caco-2 or HT-29 MTX cells. The bacteria were harvested and analyzed by flow cytometry to determine the subpopulations of GFP expressing bacteria. The dot plots identify the gate that were set to determine GFP positive (GFP+) bacterial cells. The non-GFP expressing wild-type *S*. Typhimurium strain in contact with polarized IEC was used to evaluate background fluorescence. The bar graph shows the average percentages ± standard deviation of GFP positive bacterial cells derived from three independent experiments. Statistical analyses were implemented with ordinary one-way ANOVA and Bonferroni´s multiple comparisons test P>0.05: ns; P≤0.05: *; P≤0.01: **; P≤0.001: ***.
(TIF)

S4 Fig Propidium iodide (PI) staining of bacteria grown in bacterial culture permissive or non-permissive for ChiA expression and secretion. *S*. Typhimurium wild-type and mutant strain Δ*stm0016* (peptidoglycan hydrolase), both carrying chromosomally encoded 3xFLAG-epitope-tagged ChiA, and rhamnose-inducible plasmid pTG0035 (*stm0017/chiR*) were incubated under bacterial culture conditions permissive for ChiA secretion (+Rha) or non-permissive (-Rha). After cultivation for 24 h in medium TTIM supplemented with or without 0.001% rhamnose to induce ChiA expression and secretion, 100 μl of the bacterial culture were stained with PI solution. The fluorescence intensities were measured using flow cytometry. Displayed is the proportion (percentage) of PI-positive bacteria (dead bacteria) compared to the total pool of analyzed bacteria. Statistical analyses were implemented with ordinary one-way ANOVA and Bonferroni´s multiple comparisons test with a P value of 0.1255 and a P value summary of not significant (P>0.05).
(TIF)

S5 Fig FliC expression patterns upon contact with IEC under ChiA inducing and non-inducing conditions. *S*. Typhimurium wild-type and Δ*stm0017* mutant strain were incubated with polarized Caco-2 and HT29-MTX cells for 3 h. FliC expression under ChiA inducing conditions (wild-type) and non-inducing conditions (Δ*stm0017/chiR*) were analyzed by Western blot analysis, using a monoclonal antibody against *E*. *coli* flagellin (1:1000). In addition, *stm0017/chiR* was overexpressed using a rhamnose inducible plasmid (pTG0035) in wild-type *S*. Typhimurium grown for 3 h in LB medium supplemented with 0.1% rhamnose. The induced expression of *chiR* leads to ChiA expression in LB medium as shown in Fig 2C.
(TIF)

S1 Table Bacterial strains and plasmids. (DOCX)

S2 Table Primer used for cloning. (DOCX)

S3 Table Primer used for (q)RT-PCR. (DOCX)
