# Supplementary figures and images for: Chitinase A, a tightly regulated virulence factor of Salmonella enterica serovar Typhimurium, is actively secreted by a Type 10 Secretion System
Source: PLoS Pathog. 2023 Apr 5;19(4):e1011306. doi: 10.1371/journal.ppat.1011306 (PMC10109510; doi:10.1371/journal.ppat.1011306)

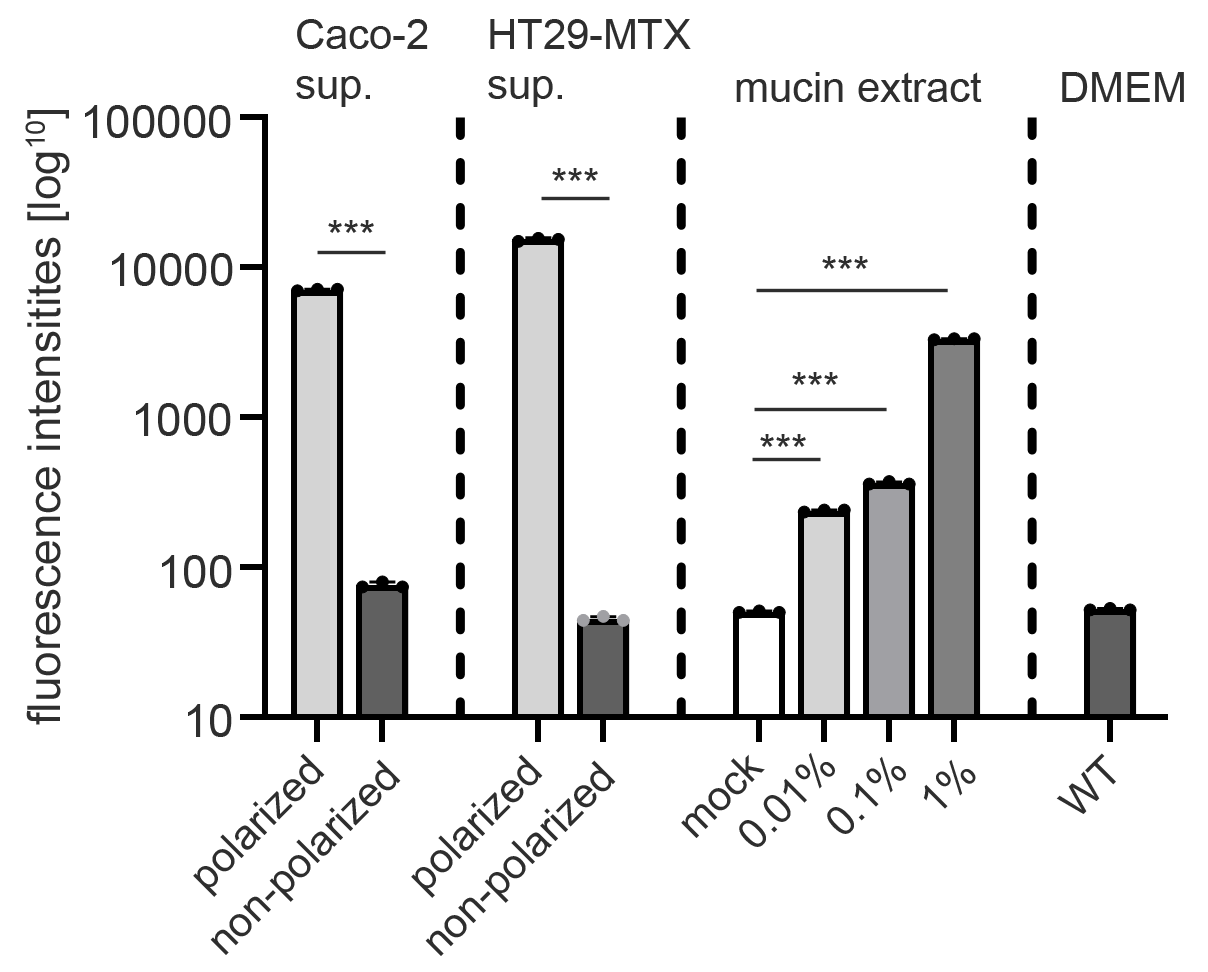

Supplement: S1 Fig — S. Typhimurium fluorescence reporter strain, carrying a chromosomal replacement of chiA::sfGFP, was incubated for 3 h in filtered cell-free supernatants of polarized and non-polarized IEC, and in various concentrations of mucin extracts (Sigma) as indicated. As a negative control, the same strain was grown in DMEM. The fluorescence intensities were normalized to equal bacterial numbers of 1x10^8^ bacteria/ml. Therefore, bacterial CFU counts were determined by plating dilution series on LB Agar plates. Statistical analysis was implemented with ordinary one-way ANOVA and Bonferroni´s multiple comparisons test P≤0.001: ***. (TIF) [file ppat.1011306.s001.tif]

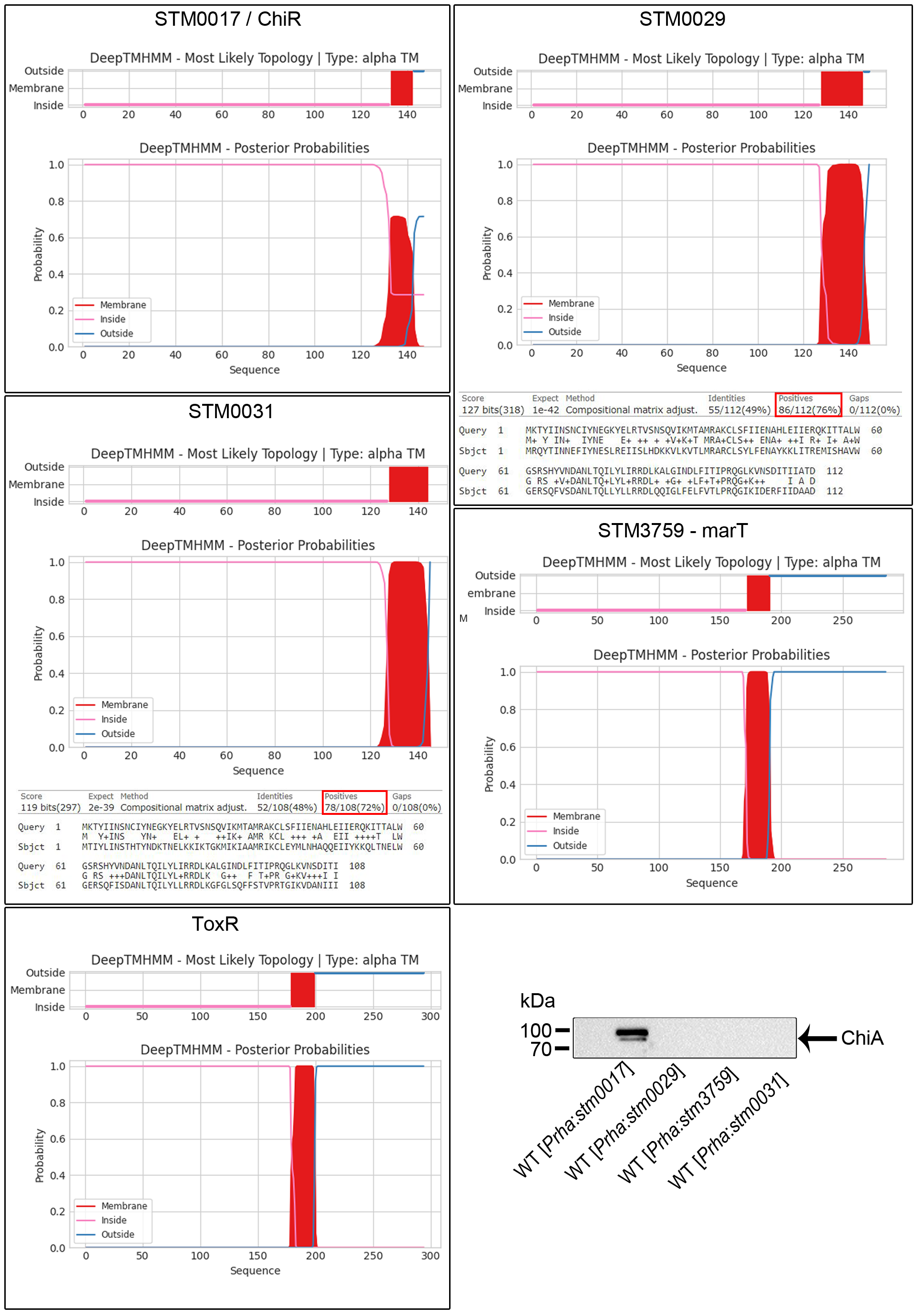

Supplement: S2 Fig — By using the Basic Local Alignment Search Tool for proteins (BLASTp) of the NIH (National Library of Medicine), three homologous proteins of STM0017 (ChiR) were identified. STM0029 and STM0031 show 49% (STM0029) and 48% (STM0031) sequence identities, and have similarities of 76% and 72%, respectively (red square). For the prediction of transmembrane helices, prediction tool TMHMM-2.0 (https://services.healthtech.dtu.dk/service.php?TMHMM-2.0) was used. The proteins were searched in FASTA formats and the probabilities for transmembrane domains are indicated as plots. The three homologous regulators were overexpressed using rhamnose inducible plasmids in a S. Typhimurium strain that expresses chromosomally integrated 3xFlag-tagged ChiA. The Western blot analysis showed that none of the tested homologues of ChiR (STM0017) led to ChiA expression. (TIF) [file ppat.1011306.s002.tif]

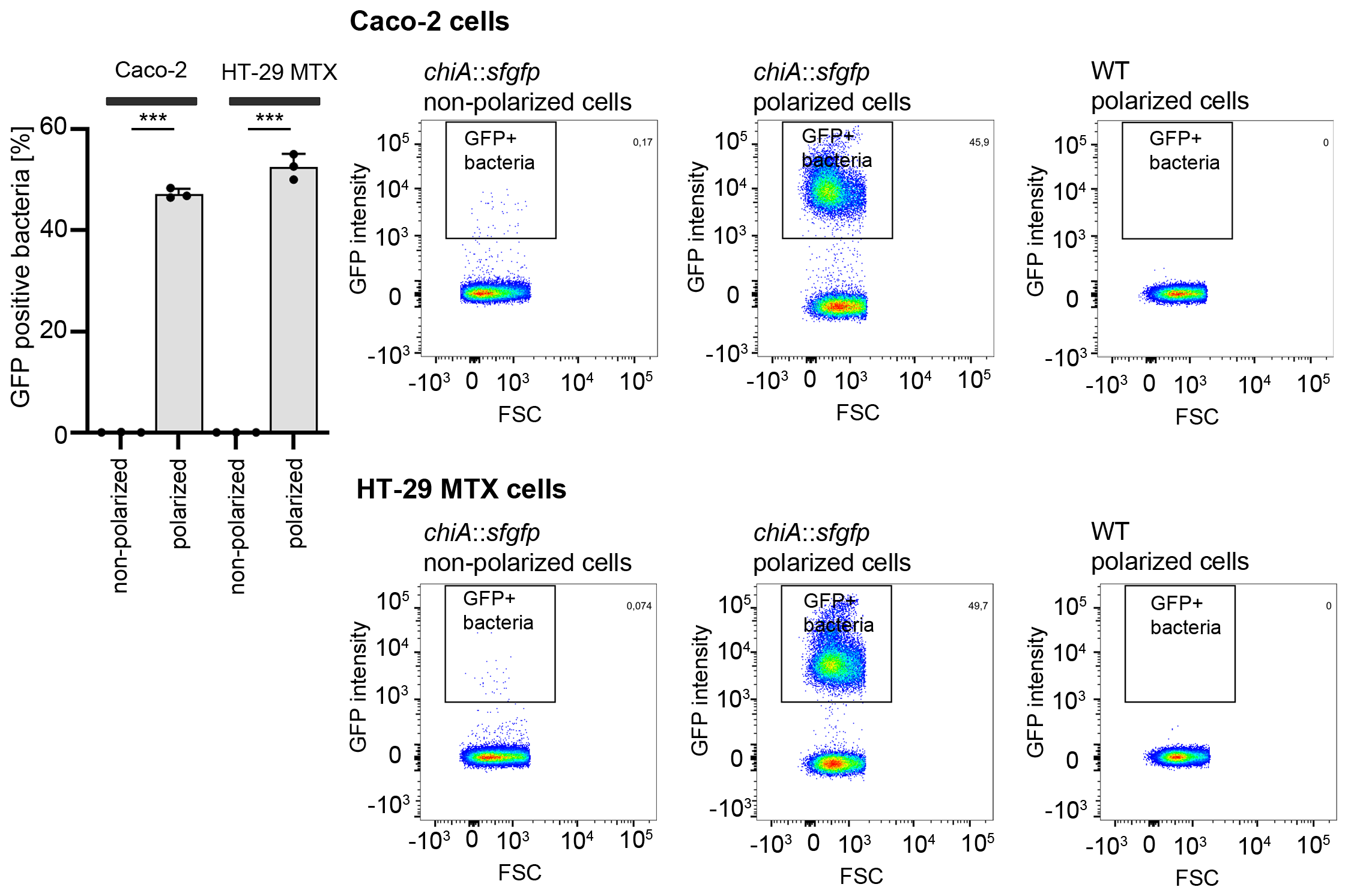

Supplement: S3 Fig — The indicated S. Typhimurium fluorescence reporter strains with chromosomal replacements chiA::sfGFP were incubated for 3 h in contact with polarized and non-polarized Caco-2 or HT-29 MTX cells. The bacteria were harvested and analyzed by flow cytometry to determine the subpopulations of GFP expressing bacteria. The dot plots identify the gate that were set to determine GFP positive (GFP+) bacterial cells. The non-GFP expressing wild-type S. Typhimurium strain in contact with polarized IEC was used to evaluate background fluorescence. The bar graph shows the average percentages ± standard deviation of GFP positive bacterial cells derived from three independent experiments. Statistical analyses were implemented with ordinary one-way ANOVA and Bonferroni´s multiple comparisons test P>0.05: ns; P≤0.05: *; P≤0.01: **; P≤0.001: ***. (TIF) [file ppat.1011306.s003.tif]

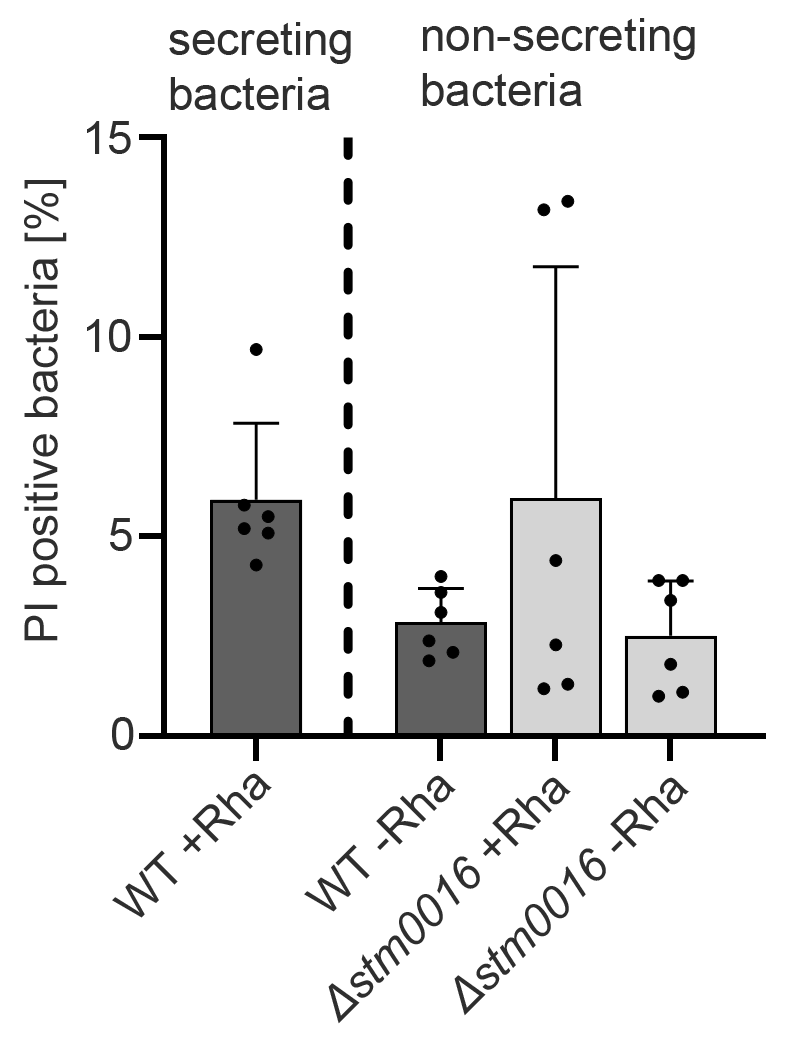

Supplement: S4 Fig — S. Typhimurium wild-type and mutant strain Δstm0016 (peptidoglycan hydrolase), both carrying chromosomally encoded 3xFLAG-epitope-tagged ChiA, and rhamnose-inducible plasmid pTG0035 (stm0017/chiR) were incubated under bacterial culture conditions permissive for ChiA secretion (+Rha) or non-permissive (-Rha). After cultivation for 24 h in medium TTIM supplemented with or without 0.001% rhamnose to induce ChiA expression and secretion, 100 μl of the bacterial culture were stained with PI solution. The fluorescence intensities were measured using flow cytometry. Displayed is the proportion (percentage) of PI-positive bacteria (dead bacteria) compared to the total pool of analyzed bacteria. Statistical analyses were implemented with ordinary one-way ANOVA and Bonferroni´s multiple comparisons test with a P value of 0.1255 and a P value summary of not significant (P>0.05). (TIF) [file ppat.1011306.s004.tif]

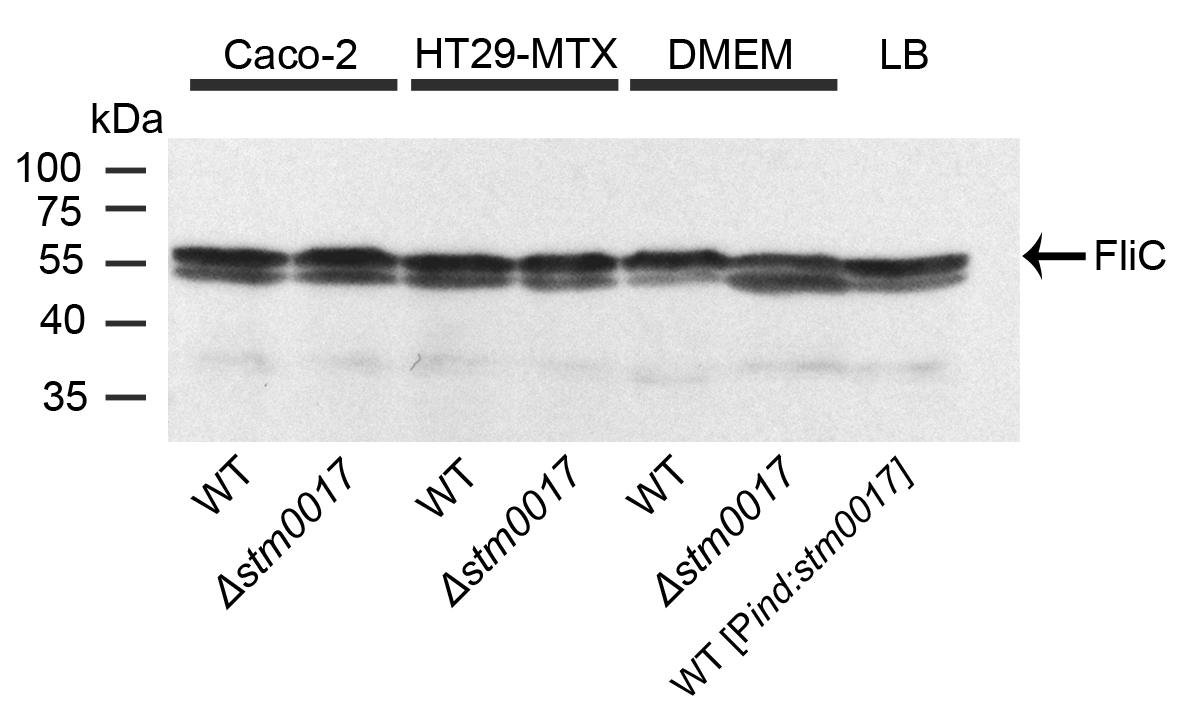

Supplement: S5 Fig — S. Typhimurium wild-type and Δstm0017 mutant strain were incubated with polarized Caco-2 and HT29-MTX cells for 3 h. FliC expression under ChiA inducing conditions (wild-type) and non-inducing conditions (Δstm0017/chiR) were analyzed by Western blot analysis, using a monoclonal antibody against E. coli flagellin (1:1000). In addition, stm0017/chiR was overexpressed using a rhamnose inducible plasmid (pTG0035) in wild-type S. Typhimurium grown for 3 h in LB medium supplemented with 0.1% rhamnose. The induced expression of chiR leads to ChiA expression in LB medium as shown in Fig 2C. (TIF) [file ppat.1011306.s005.tif]
