# Supplementary material for: Chitinase A, a tightly regulated virulence factor of Salmonella enterica serovar Typhimurium, is actively secreted by a Type 10 Secretion System
Source: PLoS Pathog. 2023 Apr 5;19(4):e1011306. doi: 10.1371/journal.ppat.1011306 (PMC10109510; doi:10.1371/journal.ppat.1011306)
Supplement: S1 Table — (DOCX) [file ppat.1011306.s006.docx]

| SB300 | *S*. Typhimurium SL1344  WT Str^R^ | [1] |
| --- | --- | --- |
| TG0016 | *S*. Typhimurium SL1344 *stm0018:3xFLAG* | This study |
| TG0014 | *S*. Typhimurium SL1344  Δ*stm0016,* *stm0018*:3xFLAG | This study |
| TG0012 | *S*. Typhimurium SL1344  Δ*stm0014,* *stm0018*:3xFLAG | This study |
| TG0013 | *S*. Typhimurium SL1344  Δ*stm0015,* *stm0018*:3xFLAG | This study |
| TG0017 | *S*. Typhimurium SL1344 *stm0018*::*sfGFP* | This study |
| TG0011 | *S*. Typhimurium SL1344  Δ*stm0018* | This study |
| *E. coli* CC118 | lambda pir (*λpir*) | [2] |
| *E. coli* ß-2163 | Pi protein expression + conjugative RP4, Δnic35, ΔdapA | [3] |
| TG0015 | S. Typhimurium SL1344  Δ*stm001,7* *stm0018*:3xFLAG | This study |
| pSB890 | Suicide vector (Sucrose) via sacB expression. R6K origin | [4] |
| pWRG167 | P*_EM7_*::*sfgfp* in pWRG81, Ap^r^ | [5] |
| pTG0033 | Promoter *chiA:sfGFP.*  685 bp promoter region upstream of *chiA* (*stm0018*), in front of sfGFP in pWRG167 | This study |
| pTG0034 | (pT12) *stm0014* behind a rhamnose inducible promotor | This study |
| pTG0035 | (pT12) *stm0017* behind a rhamnose inducible promotor | This study |
| pTG0044 | Δ*P:sfGFP*  pWRG167 with deleted PEM7 promoter in front of sfGFP | This study |
| pTG0052 | pT12 expressing *stm0029* | This study |
| pTG0053 | pT12 expressing *stm3759* | This study |
| pTG0055 | pT12 expressing *stm0031* | This study |
| pTG0069 | pT2 expressing *recA:3xFLAG* | This study |
| pTG0073 | pT12 with deleted Prhamnose, expressing *chiA (stm0018)* under native promoter (685 bp upstream) | This study |
| pSB3591 (pT12) | mid copy, CloDF13 ori, rhamnose ind. promoter | [6] |
| pSB3406 (pT10) | rhamnose ind. promoter, 3xFlag tag | [6] |
| pTACO2 (pT2) | p15Aori, low copy, rhamnose ind. promoter | [6] |
| pTG0068 | pT10 expressing *recA:3xFLAG* | This study |
| pTG0069 | pT2 expressing *recA:3xFLAG* | This study |

References

1. Hapfelmeier S, Stecher B, Barthel M, Kremer M, Muller AJ, Heikenwalder M, et al. The Salmonella pathogenicity island (SPI)-2 and SPI-1 type III secretion systems allow Salmonella serovar typhimurium to trigger colitis via MyD88-dependent and MyD88-independent mechanisms. J Immunol. 2005;174(3):1675-85. Epub 2005/01/22. doi: 10.4049/jimmunol.174.3.1675. PubMed PMID: 15661931.

2. Herrero M, de Lorenzo V, Timmis KN. Transposon vectors containing non-antibiotic resistance selection markers for cloning and stable chromosomal insertion of foreign genes in gram-negative bacteria. J Bacteriol. 1990;172(11):6557-67. Epub 1990/11/01. doi: 10.1128/jb.172.11.6557-6567.1990. PubMed PMID: 2172216; PubMed Central PMCID: PMCPMC526845.

3. Demarre G, Guerout AM, Matsumoto-Mashimo C, Rowe-Magnus DA, Marliere P, Mazel D. A new family of mobilizable suicide plasmids based on broad host range R388 plasmid (IncW) and RP4 plasmid (IncPalpha) conjugative machineries and their cognate Escherichia coli host strains. Res Microbiol. 2005;156(2):245-55. Epub 2005/03/08. doi: 10.1016/j.resmic.2004.09.007. PubMed PMID: 15748991.

4. Kaniga K, Bossio JC, Galan JE. The Salmonella typhimurium invasion genes invF and invG encode homologues of the AraC and PulD family of proteins. Mol Microbiol. 1994;13(4):555-68. doi: 10.1111/j.1365-2958.1994.tb00450.x. PubMed PMID: 7997169.

5. Bender JK, Wille T, Blank K, Lange A, Gerlach RG. LPS structure and PhoQ activity are important for Salmonella Typhimurium virulence in the Galleria mellonella infection model [corrected]. PLoS One. 2013;8(8):e73287. Epub 2013/08/21. doi: 10.1371/journal.pone.0073287. PubMed PMID: 23951347; PubMed Central PMCID: PMCPMC3738532.

6. Dietsche T, Tesfazgi Mebrhatu M, Brunner MJ, Abrusci P, Yan J, Franz-Wachtel M, et al. Structural and Functional Characterization of the Bacterial Type III Secretion Export Apparatus. PLoS Pathog. 2016;12(12):e1006071. Epub 2016/12/16. doi: 10.1371/journal.ppat.1006071. PubMed PMID: 27977800; PubMed Central PMCID: PMCPMC5158082 performed the experiments described in this paper. She is currently employed by NovoNordisk. All other authors have declared that no competing interests exist.
