# Supplementary material for: Chitinase A, a tightly regulated virulence factor of Salmonella enterica serovar Typhimurium, is actively secreted by a Type 10 Secretion System
Source: PLoS Pathog. 2023 Apr 5;19(4):e1011306. doi: 10.1371/journal.ppat.1011306 (PMC10109510; doi:10.1371/journal.ppat.1011306)
Supplement: S2 Table — (DOCX) [file ppat.1011306.s007.docx]

| upstreamchiA-2-for | CTCTGTCATTTTCTGAAACTCTTCATGCTGATAAAAAGATCGTTAATACCAAGCG |
| --- | --- |
| upstreamchiA-2-rev | TTCGCCTTTGCGCATACTTTTCTCCTCTTTCAAATTTCCTTTTACGTTTCAAAAT |
| pWRG167-for | AAAGAGGAGAAAAGTATGCG |
| pWRG167-rev | CAGCATGAAGAGTTTCAGAA |
| pT12-for | ATCCTCTAGAGTCGACCTGC |
| pT12-rev | GGTGAATTCCTCCTGAATTTCATTACG |
| pT12-stm0014-for | TTTTTAGACTGGTCGTAATGAAATTCAGGAGGAATTCACCATGGGATCGAAAGGTGCCAA |
| pT12-stm0014-rev | ACAGCCAAGCTTGCATGCCTGCAGGTCGACTCTAGAGGATTTAGCGACGGAAGGAGGAAA |
| pT12-stm0017-for | TTTTTAGACTGGTCGTAATGAAATTCAGGAGGAATTCACCATGAAAACTTATATCATTAA |
| pT12-stm0017-rev | ACAGCCAAGCTTGCATGCCTGCAGGTCGACTCTAGAGGATTTAGTGAATATGCAGATACA |
| pT12-STM0029-for | tttttagactggtcgtaatgaaattcaggaggaattcaccATGAGACAATATACTATTAA |
| pT12-STM0029-rev | acagccaagcttgcatgcctgcaggtcgactctagaggatTCAGATAAACGGCGCTAAAA |
| pT12-STM0031-for | tttttagactggtcgtaatgaaattcaggaggaattcaccATGACTATTTATTTAATTAA |
| pT12-STM3759-rev | acagccaagcttgcatgcctgcaggtcgactctagaggatTTACCCATGTGTCACCCCGC |
| pT2-for | TCGACTCTAGAGGATCC |
| pT2_new-rev | CCTCCTGAATTTCATTACGAC |
| pT2_recA3xF-for | TCGTAATGAAATTCAGGAGGAATTCACCATGGCTATCGACGAAAACAA |
| pT2_recA3xF-rev | CGGGGATCCTCTAGAGTCGATTATCATTTGTCATCGTCAT |
| pT10recA-for | AAATTCAGGAGGAATTCACCATGGCTATCGACGAAAACAA |
| pT10recA-rev | CCGTCATGGTCTTTGTAGTCAAAATCTTCGTTGGTTTCTG |
| pT12-STM0031-for2 | ggtcgtaatgaaattcaggaggaattcaccATGACTATTTATTTAATTAACAGCACGCAC |
| pT12-STM0031-rev2 | ttgcatgcctgcaggtcgactctagaggatTTATCGTATTAAATAAATAACCATTATGAC |
| pT10-rev | GGTGAATTCCTCCTGAATTTCATT |
| pSB890(2)-for | AATCTTCTTTATCGTAAAAAATGCCCTCTTGGGTTATC |
| pSB890(2)-rev | TATTTTATTTATCTTTCAAGCTCAATAAAAAGCCCCACC |
| 890stm0014-for | CTTGAAAGATAAATAAAATATATTCCGACGCTGGAGGAGT |
| 890stm0014-rev | TTTTTACGATAAAGAAGATTAGGCGGCGCCACCCATTGGG |
| stm0014del-for | TTTCCGGAGGCAGACAGCGATACACCTCATTTTTACGCTT |
| stm0014del-rev | AAGCGTAAAAATGAGGTGTATCGCTGTCTGCCTCCGGAAA |
| 890stm0015-for | CTTGAAAGATAAATAAAATATCAGGAAATGCGGCTATGGC |
| 890stm0015-rev | TTTTTACGATAAAGAAGATTGGTAAATATGAACTGAGGAC |
| stm0015del-for | AATGATTTCGGAGTGTTAAATTATCATTTGCAGTATCAGG |
| stm0015del-rev | CCTGATACTGCAAATGATAATTTAACACTCCGAAATCATT |
| pSB890stm0016del-for | AGTGAACTGCAGCCCGGGGGATCCACTAGTTCTAGAGCGGCCGGTTTCGCAACACGAGCG |
| pSB890stm0016del-rev | ATTTTATTTATCTTTCAAGCTCAATAAAAAGCCCCACCGCGTCCGGCATAGAGAATCAAA |
| stm0016del-for | CTAAGCGAAGGAAAGGGAAGACCAGGCTGAAAAAAGACGC |
| stm0016del-rev | GCGTCTTTTTTCAGCCTGGTCTTCCCTTTCCTTCGCTTAG |
| 890stm0017-for | CTTGAAAGATAAATAAAATAGAAGAGTCAGAAGCGGCGGT |
| 890stm0017-rev-new | TTTTTACGATAAAGAAGATTCTCCTTGTGTTAATTATTCA |
| stm0017del-for | CATTTCTAAAGGAAGACGTTGCGAAGGAAAGGGAAGATGA |
| stm0017del-rev | TCATCTTCCCTTTCCTTCGCAACGTCTTCCTTTAGAAATG |
| 890stm0018-for | CTTGAAAGATAAATAAAATAGAATCGCGTAGGCTTCAGCG |
| 890stm0018-rev-new | TTTTTACGATAAAGAAGATTGAAACAGCAGCTCAATCACC |
| stm0018del-for | ACGTAAAAGGAAATTTGAAATACACGGTATTAAGCCGATG |
| stm0018del-rev | CATCGGCTTAATACCGTGTATTTCAAATTTCCTTTTACGT |
| pT103xF-chiA-rev | TGATCTTTATAATCACCGTCATGGTCTTTGTAGTCGTAAGCGCCAAGATCGGT |
| pT10chiA-for | AGACTGGTCGTAATGAAATTCAGGAGGAATTCACCATGGCTACAAGCAAACTGAT |
| pT10-3XFlag-for | GACTACAAAGACCATGACGGTGA |
| pT10-rev | GGTGAATTCCTCCTGAATTTCATT |
| pSB890chiA3XFLAG-rev | ATTTTATTTATCTTTCAAGCTCAATAAAAAGCCCCACCGCTGCATCTCATCATTCGCCAG |
| pSB890chiA3XFLAGdown-for | ATGACGATGACAAATGATAATACACGGTATTAAGCCGATG |
| pSB890chiA3XFLAG-for | AGTGAACTGCAGCCCGGGGGATCCACTAGTTCTAGAGCGGATGGCTACAAGCAAACTGAT |
| pSB890chiA3XFLAGdown-rev | CATCGGCTTAATACCGTGTATTATCATTTGTCATCGTCAT |
| sfGFP-stm0018down-for | TGGATGAACTGTATAAATAATACACGGTATTAAGCCGATG |
| sfGFP-stm0018up-rev | AGTTCTTCGCCTTTGCGCATTTTCAAATTTCCTTTTACGTTTCAAAATTGTCGC |
| sfGFP-stm0018up-for | ACGTAAAAGGAAATTTGAAAATGCGCAAAGGCGAAGAACT |
| sfGFP-stm0018down-rev | CATCGGCTTAATACCGTGTATTATTTATACAGTTCATCCATGCCATGC |
