# Supplementary material for: Chitinase A, a tightly regulated virulence factor of Salmonella enterica serovar Typhimurium, is actively secreted by a Type 10 Secretion System
Source: PLoS Pathog. 2023 Apr 5;19(4):e1011306. doi: 10.1371/journal.ppat.1011306 (PMC10109510; doi:10.1371/journal.ppat.1011306)
Supplement: S3 Table — (DOCX) [file ppat.1011306.s008.docx]

| qPCR stm0017-rev | ACATAATGGCTTCGACTTCCC |
| --- | --- |
| qPCR stm0017-for | AGGACGGTCTCAAATTCACAG |
